# Relative importance of genotype, gene expression, and DNA methylation on complex traits in perennial ryegrass

**DOI:** 10.1002/tpg2.20253

**Published:** 2022-08-17

**Authors:** Marta Malinowska, Anja Karine Ruud, Just Jensen, Simon Fiil Svane, Abraham George Smith, Andrea Bellucci, Ingo Lenk, Istvan Nagy, Mattia Fois, Thomas Didion, Kristian Thorup‐Kristensen, Christian Sig Jensen, Torben Asp

**Affiliations:** ^1^ Center for Quantitative Genetics and Genomics Aarhus Univ. Slagelse Denmark; ^2^ Dep. of Plant and Environmental Sciences Univ. of Copenhagen Taastrup Denmark; ^3^ Dep. of Computer Science Univ. of Copenhagen Denmark; ^4^ Research Division DLF Seeds A/S Store Heddinge Denmark

## Abstract

The growing demand for food and feed crops in the world because of growing population and more extreme weather events requires high‐yielding and resilient crops. Many agriculturally important traits are polygenic, controlled by multiple regulatory layers, and with a strong interaction with the environment. In this study, 120 F_2_ families of perennial ryegrass (*Lolium perenne* L.) were grown across a water gradient in a semifield facility with subsoil irrigation. Genomic (single‐nucleotide polymorphism [SNP]), transcriptomic (gene expression [GE]), and DNA methylomic (MET) data were integrated with feed quality trait data collected from control and drought sections in the semifield facility, providing a treatment effect. Deep root length (DRL) below 110 cm was assessed with convolutional neural network image analysis. Bayesian prediction models were used to partition phenotypic variance into its components and evaluated the proportion of phenotypic variance in all traits captured by different regulatory layers (SNP, GE, and MET). The spatial effects and effects of SNP, GE, MET, the interaction between GE and MET (GE × MET) and GE × treatment (GE_Control_ and GE_Drought_) interaction were investigated. Gene expression explained a substantial part of the genetic and spatial variance for all the investigated phenotypes, whereas MET explained residual variance not accounted for by SNPs or GE. For DRL, MET also contributed to explaining spatial variance. The study provides a statistically elegant analytical paradigm that integrates genomic, transcriptomic, and MET information to understand the regulatory mechanisms of polygenic effects for complex traits.

## Core Ideas

- Yield, quality, and deep root length were screened in a semifield, state‐of‐the‐art root phenotyping facility.
- This study integrates genomic, transcriptomic, and MET information to better understand complex traits.
- Bayesian regression models fit high‐dimensional data and account for interactions between and within omics layers.
- By combining multiple omics layers, it is possible to capture a larger part of phenotypic variation.

**Abbreviations**
CEL: cellulose content
DIC: deviance information criterion
DMDig: dry matter digestibility
DRL: deep root length
DW: dry matter weight
GBS: genotyping‐by‐sequencing
GE: gene expression
MET: DNA methylomic
QTL: quantitative trait loci
RNA‐seq: RNA sequencing
SNP: single‐nucleotide polymorphism

## 1 INTRODUCTION

Events of extreme weather fueled by climate change already have adverse effects on crop production (Lesk et al., 2016). In Denmark, fewer but heavier rainfalls during summer and more frequent heat waves of longer duration are predicted to challenge agricultural production (The Danish Environmental Protection Agency, 2020). Perennial ryegrass (*Lolium perenne* L.) is the predominant forage grass in temperate climates, including Denmark, because of its rapid growth and high forage quality, but growth and yield are challenged by such weather extremes (Buttler et al., 2019; Cheplick et al., 2000; Kemesyte et al., 2017). Hence, we need a deeper understanding of the biological processes and molecular mechanisms behind the responses to different unfavourable events like excess or limited water.

According to The Central Dogma (Crick, 1970, 1958), the genome is the basal information layer of the cell, static by nature, and does not directly provide information about the biological mechanisms it encodes. Downstream from the genomic layer, dynamic functional omics layers provide high‐dimensional endophenotypes (intermediate phenotypes) and potential sources of information. The transcriptome and proteome are directly encoded in the genome and modulated by environmental influences, whereas the shape of the metabolome, and largely the epigenome, are products of proteome functionality and environmental signals, respectively (Haas et al., 2017). Many biological processes are regulated by interactions between the genome and downstream regulatory layers (Haas et al., 2017), omic × omic and omic × environment interactions, thus serving as modulators of genetic and environmental effects on the final phenotype, that is, they can be seen as intermediate phenotypes.

Because of increased availability and decreasing prices of next‐generation sequencing, combined with fewer constraints because of computational power, there is a growing interest in the potential of downstream omics data to bridge the gap between genotype and phenotype. Several studies have used omics data as intermediate phenotypes, most notably in gene expression (GE) quantitative trait loci (QTL) and metabolic QTL mapping. Such studies aim to identify candidate genes, diagnostic markers, or genetic elements underlying complex phenotypes in crop plants as reviewed in Druka et al. (2010). The DNA methylation (MET) patterns could explain up to 65% of the phenotypic variance for plant height in an *Arabidopsis thaliana* (L.) Heynh. isogenic epirecombinant inbred line population, only segregating for wild type and mutant DNA methyltransferase *ddm1‐2* (Hu et al., 2015).

Particularly, downstream omics data has the potential to capture nonadditive genetic effects including epistasis. The statistical meaning of epistasis is defined as deviations from additivity in a statistical model, whereas the biological meaning is the variations resulting from gene actions (Fisher, 1918). Although epistatic models based on low marker density have been shown to increase prediction accuracy compared with additive models (Schrauf et al., 2020), other authors have found no (Jarquín, Kocak, et al., 2014) or a negative (Lorenzana & Bernardo, 2009) effect of epistasis. To detect significant gain in predictive ability by including marker‐based epistasis requires a large population size and depends on the genetic relationships within the population. For example, Raffo et al. (2022) observed a significant increase in predictive ability compared with an additive genetic model using leave‐one‐line‐out cross‐validation in >2,000 winter wheat (*Triticum aestivum* L.) breeding lines. Using a leave‐one‐breeding‐cycle‐out scheme, where close relatives like full‐sibs were left out, no improvement was detected, which the authors attribute mainly to the influence of relationships among the individuals in the reference and validation populations (Raffo et al., 2022). Meanwhile, integrating endophenotypes in the prediction models can contribute to biological explanations of epistatic and other interactions (Civelek & Lusis, 2014).

**Core Ideas**

- Yield, quality, and deep root length were screened in a semifield, state‐of‐the‐art root phenotyping facility.
- This study integrates genomic, transcriptomic and MET information to understand complex traits better.
- Bayesian regression models fit high‐dimensional data and account for interactions between and within omics layers.
- By combining multiple omics layers, it is possible to capture a larger part of phenotypic variation.

The phenotype is the result of the interactions between an organism's genotype and the environment. Because of such interactions, predicting genetic breeding values across environments can be challenging. Nevertheless, predicting the phenotype from genotypic and environmental information is of utmost interest in medicine and crop breeding (Grinberg et al., 2020), for example, by accounting (Svane, Dam, et al., 2019; Svane, Jensen, et al., 2019) for environmental risk factors in the prediction of disease outcome or development of obesity (Huang & Hu, 2015) or the performance of a crop cultivar in different environments (van Eeuwijk et al., 2019). Adding secondary data to explain genotype × environment (G × E) interactions has become a focus in plant breeding in recent years, increasing prediction accuracy and predicting performance in new environments (Jarquín, Crossa, et al., 2014; Malosetti et al., 2016; Robert et al., 2020). Downstream regulatory omic layers, which are intermediate phenotypes influenced by both genetic effects and the environment, are examples of such secondary data.

Clustering methods based on single omics have been shown to successfully group, for example, subtypes of breast cancer in humans (Sørlie et al., 2001). By adding whole‐genome transcriptome profiles in Bayesian prediction models, Vazquez et al. (2016) demonstrated that increases in predictive ability of breast cancer outcome were not due to such subtype clustering but to other patterns in the gene expression data. Whole‐genome methylation profiles offered a higher prediction power and accuracy than any commonly used covariates for predicting breast cancer survival (Vazquez et al., 2016).

Predicting performance and combining abilities in hybrids usually achieves relatively low predictability from parental genotype and phenotype, and heterosis is a product of complex gene × gene interactions and epigenetics (Zenke‐Philippi et al., 2016). Thus, there have been many efforts to include soluble RNA, messenger RNA, and metabolites in hybrid performance prediction models in maize (*Zea mays* L.) (Fu et al., 2012; Schrag et al., 2018; Westhues et al., 2017; Zenke‐Philippi et al., 2016) and rice (*Oryza sativa* L.) (Wang et al., 2019). For example, Dan et al. (2016) showed that metabolites provided high predictive abilities for predicting hybrid performance in rice using partial least square regression.

Azodi et al. (2020) developed prediction models trained on transcriptome data from the maize pangenome population (diverse inbred lines) to understand genetic mechanisms for flowering time, plant height, and yield. They found that genetic markers and transcriptome data captured different aspects of the phenotypic variation and that transcriptomes from the seedling stage (Hirsch et al., 2014) could predict phenotypes at the adult stage better than a baseline model using population structure with the first five principal components of genetic marker basis. However, the prediction of additive genotype based on genetic marker data and models, including transcript level data for phenotype prediction, were not compared appropriately.

Under field and semifield conditions, plants can experience heterogeneous conditions with variable spatial and temporal patterns. The plants’ responses to combinations of stresses often have nonadditive effects on the phenotypes (Atkinson & Urwin, 2012; Cruz et al., 2020; Rasmussen et al., 2013; Thoen et al., 2017). Omics data generated under field conditions is noisier than data collected in controlled environments (Schrag et al., 2018). Despite the challenges, data from field‐grown plants is essential for closing the gap between field and lab (Alexandersson et al., 2014; Cruz et al., 2020; Nelissen et al., 2020). Cruz et al. (2020) investigated the spatial autocorrelation for phenotype, metabolite, and GE data sampled across a field of the inbred maize line B104. They suggested that patterns of spatial autocorrelation among metabolites and transcripts, and part of the variability observed among individual plants, are due to microenvironmental factors with a spatial structure. In prediction models, such spatial effects should be disentangled from the true genetic effects in the omics data. Ignoring interactions with the genotype, including complex environmental–microenvironmental factors, can lead to biased estimates of residual variance and single‐nucleotide polymorphism (SNP) heritability (Ni et al., 2019). González‐Reymúndez et al. (2017) found that the effects of omic × treatment interactions explained variance in human breast cancer survival time not captured by clinical covariates.

In this study, we investigated the role and relative importance of genomic (SNP), transcriptomic (GE), and MET data to predict the yield, quality, and deep root length (DRL) of 120 F_2_ families of perennial ryegrass. The plants were grown across a water gradient in a semifield facility with controlled subsoil irrigation (Svane, Jensen, et al., 2019) and phenotyped for three feed quality traits that can be affected by water availability. Root growth traits are important for drought tolerance, and DRL measured with multispectral imaging was included. We computed a *K*‐nearest weighted spatial connectivity matrix to capture spatial effects. We then ran a sequence of Bayesian models, implemented in the BGLR package (Pérez & de los Campos, 2014), to partition the variance explained by spatial effects, omics, omic × omic interaction, and GE × treatment interactions. We further investigated the effects of the individual and different combinations of sets of predictors on prediction accuracy. By this effort, we were able to partition the genetic, spatial, and environmental components of the GE and methylation layers. To the best of our knowledge, neither treatment × omic interactions, nor the influence of spatial effects in omics‐augmented prediction models with different genotypes, have been investigated in plants.

## 2 MATERIALS AND METHODS

### 2.1 Plant material and experimental design

**FIGURE 1:**
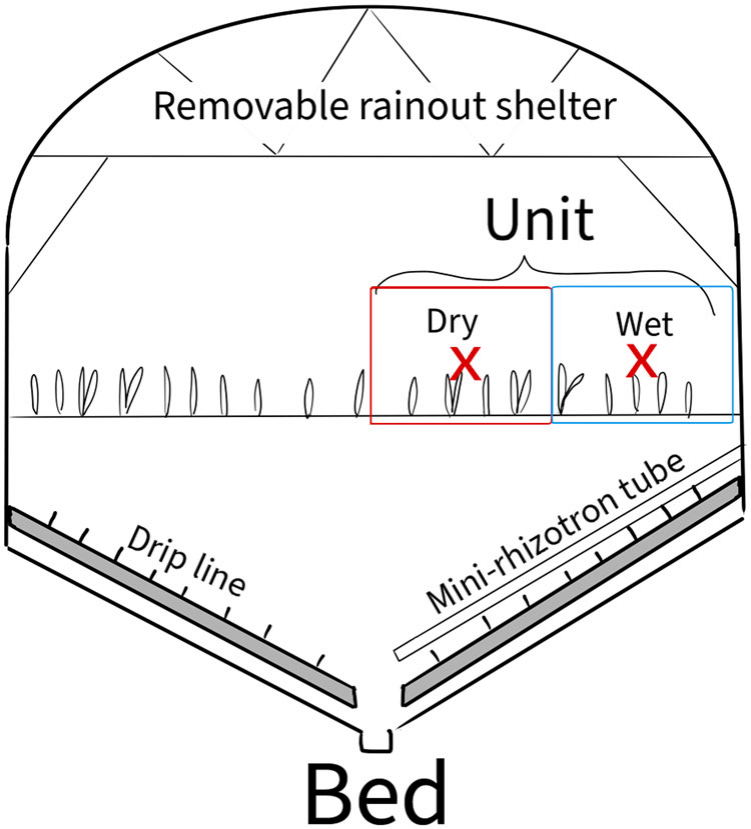
Cross‐section of a bed in RadiMax, with division in experimental units and specification of drought (Dry) and control (Wet) parts of the unit. The red ‘X’ represents approximately where, within each family or row, the sampling for omics data were collected. Each F_2_ family was grown in rows across two units, that is, from the center to the edge of the facility. Sampling for transcriptomic and methylomic data was done from the unit equipped with minirhizotron tubes. Water was controlled by the subsurface irrigation lines, where plants toward the center of the facility had to grow deeper roots to reach water. The facility is described in detail in Svane, Jensen, et al. (2019) and the root imaging set up in Svane, Dam, et al. (2019)

The experiment was conducted in RadiMax (Svane, Jensen, et al., 2019), a semifield root phenotyping facility located at Copenhagen University's experimental farm (Taastrup, Denmark) in 2017. The trial was conducted in two separate beds (hereafter referred to as Bed 3 and Bed 4), each divided into half‐beds (one half‐bed is an experimental unit, see Figure 1). Each bed is operated with independent subsurface water management systems (Figure 1) and has a V‐shaped concrete bottom with soil depth increasing from 0.8 to 2.1 m in each unit (maximal depth: 2.1 m; slope: 15.8°; 18 subirrigation tubes). The subsurface irrigation system was described in detail by Svane, Jensen, et al. (2019). In brief, driplines were placed along the bottom of each unit at the 0.20‐m interval. Perforated pipes leaked water flowing by gravity, whereas the capillary soil movement provided irrigation to the plants. Subirrigation was controlled by the sensor system monitoring the water uptake (Supplemental Figure S1). Because of the water availability gradient created by a depth to the subsoil irrigation system and movable rain‐out shelters, plants growing toward the deeper part of the unit had access to limited amounts of water and were expected to exhibit drought stress responses (called ‘drought’ treatment for simplicity). Units are fitted with minirhizotrons along one sloping bottom, allowing for root observation at various soil depths along half of each row (or an experimental unit). One‐hundred twenty diploid F_2_ families of forage type perennial ryegrass were randomly assigned to each of Beds 3 and 4 sown in October 2016 with a planting density of 2 gm^–2^ (0.5 gm^–1^ with 25 cm between the rows) and grown for 1 year in the facility. The plants were grown per F_2_ family in rows (9.7 m long) across two units directly above the minirhizotrons tubes with a 25‐cm distance between each row. Thus, a unique row number also refers to a unique F_2_ family grown in that row. In this experiment, only half of the row that was located above the minirhizotron tubes were considered (Figure 1), that is, one entire unit per bed.

### 2.2 Phenotypic records

The grass was cut three times during the season. All cuts were carried out at 6 cm aboveground level with a Nibbi Brik 3 walk‐behind tractor with cutter bar ESM 1.22 mt (NIBBI). Each row was divided into four subsamples at harvest—two from the deep midsections (‘drought’ treatment) and two from the border sections (‘control’)—and analyzed separately for yield‐ and quality‐related phenotypes. As samples for RNA and methylomic data were collected from one unit per F_2_ family, we have only included phenotypic data from the same unit (Figure 1), that is, two data points per F_2_ family. The quality measurements were done by DLF Seeds (Store Heddinge, Denmark) using protocols for the ANKOM 2000 fiber analyzer (ANKOM Technology). The third cut was chosen for data collection, including sampling of the omics data, because of the timing and expected effect of the drought treatment. Three aboveground phenotypes were included: dry matter weight (DW) of the third cut, cellulose content (CEL), and dry matter digestibility (DMDig) in percentage of total DW. For DW, grass samples were stored for a maximum of 1 h in a cool environment and dried at 60 °C in a hot‐air circulating oven until no further weight loss could be measured (24–40 h of drying). Then, the dried samples were milled in a Retsch SM 300 cutting mill to 1‐mm particle size, and, after mixing, samples were subjected to chemical analysis.

Cellulose content (in percentage of dry matter) was estimated based on the acid detergent fiber (ADF) (ANKOM Technology, 2017a) and acid detergent lignin (ADL) (ANKOM Technology, 2022) values in percentage of dry matter. In principle, the cellulose content was described as the ADF content minus ADL (CEL = ADF − ADL), all based on percentage dry matter.

Dry matter digestibility was based on the neutral detergent fiber (ANKOM Technology, 2017b), which represents the cellulose, hemicellulose, and lignin part of the cell wall. After neutral detergent fiber analysis, the cell wall samples were treated with a cellulase mix (CELLULASE R‐10 from Duchefa Biochemistry), and the indigestible neutral detergent fiber, neutral cellulase fiber (NCF), was measured by weighing (percentage of dry matter) (ANKOM Technology, 1998) using a modified protocol without gamanase. The DMDig is the fraction of the cell wall not digestible by the cellulase enzyme mix after 40 h of incubation at 39 °C. It is assumed that everything else is 100% digestible so that DMDig% = 100% − NCF%.

A total of 78,200 root images were taken with the multispectral camera system as described in Svane, Dam, et al. (2019) on four dates in 2017. These dates were the 10 May, 29 May, 6 July, and 22 August. The first imaging coincided with the beginning of the growing season, and the remaining three dates corresponded to a regular cutting regime under Danish growth conditions and the increasing drought stress, as assessed by a sensor network consisting of volumetric water content and temperature sensors (Acclima, Inc.) (Svane, Jensen, et al., 2019). The images were taken of roots through minirhizotron tubes {acrylic glass [poly(methyl methacrylate)]} installed beneath each row at a 15° angle to the horizontal, allowing imaging from 0.4 to 1.8 m depth. Pseudo‐RGB images, generated using the red (660 nm), green (590 nm) and blue (450 nm) waveband from all four dates, were used for segmentation model training with RootPainter (Smith, Han, et al., 2022). From these 78,200 images, 4,000 were selected at random using the ‘create training dataset’ functionality from RootPainter with a target size specified as 900 × 900. In short, a U‐Net based convoluted neural network system in Python (v3.6.4.) using PyTorch (Paszke et al., 2017) was used to segment the root images. The first 450 images were annotated correctively with attention to detail as described in Smith, Han, et al. (2022). The subsequent 1,350 images were inspected at a faster pace to identify larger error regions or outliers, which would then be annotated correctively. Model training was then stopped, and all 78,200 images were segmented. Then, root length was extracted from the generated segmentations via skeletonization and pixel counting (Smith, Petersen, et al., 2020). The interactive model training, including annotation, model training, and reviewing model predictions, took 5.5 h using two NVIDIA RTX 2080 Ti GPUs. Segmenting all 78,200 images took ∼1 d. In total, 22,175 images from 6 July were used for this study. Of the 300 tubes, image data from eight tubes were missing because of overheating failure of the camera computer and operator error during the day of imaging. The extracted root length from the segmentation was correlated to a manually curated grid count of 200 randomly chosen and not annotated images from the data set (Supplemental Figure S2), giving a *R* ^2^ value of .85.

Furthermore, six tubes were excluded because of bent minirhizotrons preventing the camera from reaching the deepest sections of the tubes. The DRL root profile trait was then computed from individual root image length measurements from 6 July using 1/470 pixels cm^–1^ as a calibration factor. The DRL was computed as the sum of root length (cm) below a cut‐off soil depth of 110 cm. We based the length of 110 cm as the depth the average intensity in the dataset started to decline, thus we are only interested in the difference in length in the deeper part of the root system. For aboveground measurements, two phenotype data points were obtained per family, one from ‘drought’ and one from ‘control’ plants (Figure 1), whereas for root length, only one measurement per family was considered for further analysis, that is, based on the sum of roots 110 cm below the soil surface. In other words, we looked at the total amount of roots below 110 cm as a phenotype not the root length in a specific location in the facility.

### 2.3 Omics data

Omics data included SNPs from combined genotyping‐by‐sequencing (GBS) and transcript‐based SNPs from the RNA sequencing (RNA‐seq) data, GE profiles from RNA‐seq, and reduced‐representation MET. Leaf samples for total GE and MET analyses were collected in RadiMax, coinciding with the third cut and sampling of material from which the aboveground phenotypes were also derived. For each F_2_ family, samples consisting of 15–20 leaves were taken from two areas (‘drought’ and ‘control’), generating two records per family. The samples were snap frozen in liquid N, ground in a Geno/Grinder 2000 (SPEX CertiPrep), and the ground tissue distributed for MET and RNA extractions.

To obtain methylomic data, epiGBS libraries were prepared as previously described (Malinowska et al., 2020) and sequenced on the Illumina HiSeq4000 platform (2 × 150 bp) by Beijing Genomics Institute (Shenzhen, China). The sequencing data were processed using the WellMeth pipeline (Malinowska et al., 2020). Demultiplexed and trimmed reads were mapped to the perennial ryegrass pseudo‐chromosome assembly (Nagy et al., 2022). The identification of MET sites was followed by polymerase chain reaction duplicate removal. The methylation level of each cytosine covered by the analysis was calculated as a proportion of methylated cytosine (#C) to sequencing depth of a position (methylated and unmethylated #T) [#C/(#C + #T)]. Single‐base DNA methylation values ranged between 0 (not methylated) and 1 (completely methylated).

The RNA was extracted with the Sigma Aldrich Total Plant RNA kit, following their standard protocol for normal leaf tissues. Gene expression profiles were obtained using RNA‐seq technology sequenced on the Illumina HiSeq4000 platform (2 × 100 bp, ∼20 M reads per sample) by the Beijing Genomics Institute (Shenzhen, China). Transcript abundance was calculated with KALLISTO (Bray et al., 2016). The HISAT2 software (Kim et al., 2019) was used to align the transcripts to the perennial ryegrass reference genome (Nagy et al., 2022). Samtools (Li et al., 2009) was used to add read groups and mark and delete duplicates. Finally, SNPs were called with freebayes (Garrison & Marth, 2012) with settings set to minimum mapping quality of 30 and minimum coverage of eight.

The GBS sampling and library preparation was performed according to Byrne et al. (2013) and Elshire et al. (2011). Briefly, leaf material for GBS data was sampled from ∼100 seedlings per F_2_ family grown on Rockwool blocks. Genomic DNA extracted from these samples was digested using the ApeKI (5‐bp recognition site) and PstI (6‐bp recognition site) restriction enzymes and sequenced on an Illumina HiSeq4000 platform. Genetic variants were called using GATK v3.7 and filtered on a mapping quality of 30.

The SNP datasets from RNA‐seq and GBS sequencing were merged based on physical position and family. The 10,432 SNPs that were found to be in common between the two datasets were merged based on their allele frequencies, that is, DNA fragments carrying alternative and reference alleles were summed prior to allele frequency (AF) calculation. Afterward, SNPs were filtered based on sequencing depth >10, SNP missingness <50%, and minor allele frequency >0.05). Finally, family AF for each biallelic SNP was extracted as the sequencing depth for DNA fragments carrying the alternative base divided by total sequencing depth. Thus, numeric values ranged between 0 and 1. Missing data points were replaced by mean AF per SNP.

Initially, variant datasets included 192,814 and 1,787,068 SNPs for GBS and RNA‐seq experiments, respectively. These were merged and filtered as described above to obtain 357,217 SNPs that were finally converted into AFs. A total of 139,003 transcripts were identified by KALLISTO (Bray et al., 2016). After filtering out transcripts with zero expression and median transcript per million (TPM) <1, log_2_(TPM + 1) values for 22,602 transcripts were included in the analysis. The MET data were filtered based on a minimum read coverage of three and a maximum of 50% missing values per sample. If a family was removed in one dataset (control or drought), it was also removed in all the other available data. A total of 276,962 unique MET positions were kept for further analysis. The remaining missing methylation values (31% of all values) were replaced with average methylation per site over all samples.

### 2.4 Spatial distribution characteristics

Moran's eigenvector maps were derived from *K*‐nearest weighted spatial connectivity matrix with R packages adespatial (Dray et al., 2018) and ade4 (Dray & Dufour, 2007). The assumed distance between two neighboring rows equals one. The spatial information was used to compute the spatial autocorrelation of analyzed traits. Moran's coefficient estimates the strength of interdependence between each phenotypic score by comparing the value of *x_i_* at location *i* with the value *x_j_* at all other locations (Zhou and Lin, 2016). In other words, spatial autocorrelation expresses a correlation in a signal among neighboring locations in multidirectional space and evaluates whether the pattern is clustered, dispersed, or random. The spatial effects were evaluated on a by‐row basis, that is, covering both drought and control treatment for each sample. Thus, the spatial effects are independent of treatment and within‐F_2_ family genetic effects.

### 2.5 Statistical models

A genomic relationship matrix for additive effects was calculated based on VanRaden (2008) modified to include family instead of individual AF (Fè et al., 2015). The AF matrix **M** was centered by mean AF per SNP and multiplied by its transposed **M′** and scaled by a constant.

The formula applied was as follows:

(1) $$G=\frac{{\mathrm{MM}}^{\prime }}{0.25\sum {\bar{F}}_{j}{\left(1-\bar{F}\right)}_{j}},$$

where the scale parameter 0.25 is due to the presence of four alleles in F_2_ families. *F̅_j_* refers to average AF for the marker *j*. Furthermore, **G** matrix diagonal elements were corrected for extra binomial variance because of reduced sequencing depth as derived by Cericola et al. (2018) for Equation 2:

(2) $$D_{Ci}=D_{Oi}\left(1-\frac{1}{{\bar{\mathrm{SD}}}_{i}+1}\right),$$

where a corrected diagonal element *D*_C_ *_i_* for every *i*th individual is obtained from the initial value *D*_O_ *_i_* transformed based on individual mean sequencing depth ${\bar{SD}}_{i}$. Only diagonal elements are corrected because off‐diagonal elements are not biased by sequencing depth because of the independence of sequencing depth of different loci.

Similarity matrices for gene expression and DNA methylation data were computed following the basic formula:

(3) $$K=\frac{{\mathrm{XX}}^{\prime }}{p}$$

where **X** is a matrix (centered and scaled) of considered features, *p* is the number of features (transcripts or MET sites).

A total of seven prediction models (Table 1) were evaluated based on data from all families grown under two treatments. Aside from three omics effects, the effect of the water availability (treatment), the spatial effect was included to account for micro‐ and macroenvironmental differences (like soil compactness, nutrient availability, shading. or location in the field) observed across units. The baseline model (Equation 4) defined the response of observed phenotypes (*y*; *N* = 240) and included treatment effect (*T_i_*) assigned a flat Gaussian prior with mean zero and variance equal to 1 × 10^10^, plus the normal, independent, and identically distributed random effects such as $l_{i}∼\mathrm{NIID}\left(0,\sigma_{l}^{2}\right)$, $\varepsilon_{\mathrm{ij}}∼\mathrm{NIID}\left(0,\sigma_{\varepsilon }^{2}\right)$, and multivariate normal prior distribution $s_{i}∼\mathrm{MVN}\left(0,K_{s}\sigma_{s}^{2}\right)$, where $\sigma_{l}^{2}$, $\sigma_{s}^{2}$, $\sigma_{\varepsilon }^{2}$ are family, spatial neighborhood, and residual variances, respectively.

(4) $$\mathrm{Baseline}\;\;:\;\;y_{\mathrm{ij}}=\mu +T_{i}+l_{i}+s_{i}+\varepsilon_{\mathrm{ij}}$$

**TABLE 1:** Main effect and interaction of the seven models used to fit the data set

Model	SPAT	FAMILY	SNP	MET	GE	MET × GE	GE_C_	GE_D_	ε
M1	X	X							X
M2	X	X	X						X
M3	X	X	X	X					X
M4	X	X	X		X				X
M5	X	X	X	X	X				X
M6	X	X	X	X	X	X			X
M7	X	X	X	X	X	X	X	X	X

*Note*. GE, transcriptomic effect; GE_C_, trancriptomic × control effect; GE_D_, transcriptomic‐by‐drought treatment effect; MET, methylomic effect; MET × GE, methylation‐by‐expression effect; SNP, genetic additive effect; SPAT, spatial effect.

Subsequently, we extended the baseline model by sequentially adding omic information (Table 1). The full model, including all omics and interaction terms is defined in Equation 5:

(5) $$\begin{array}{ccc} y_{ij} & = & \mu +T_{i}+l_{i}+s_{i}+g_{i}+u_{ij}+m_{ij}+um_{ij}+u_{Ci}\\ & & +\;u_{Di}+\varepsilon_{ij} \end{array}$$

Where baseline model was extended by random effects of markers (SNP) with a multivariate normal prior distribution $g_{i}∼\mathrm{MVN}\left(0,G\sigma_{g}^{2}\right)$ followed by genomic main effects associated with GE, $u_{ij}∼\mathrm{MVN}\left(0,K_{u}\sigma_{u}^{2}\right)$; MET, $m_{ij}∼\mathrm{MVN}\left(0,K{\sigma_{m}}_{m}^{2}\right)$, as well as GE under both levels of water availability either when drought (GE_D_) was applied $u_{Di}∼\mathrm{MVN}\left(0,K_{\mathrm{ud}}\sigma_{\mathrm{ud}}^{2}\right)$ or not (GE_C_) $u_{Ci}∼\mathrm{MVN}\left(0,K_{\mathrm{uc}}\sigma_{\mathrm{uc}}^{2}\right)$. Therefore, **G** was a marker‐derived genomic relationship matrix as calculated in Equation 1 and $\sigma_{g}^{2}$ was genomic variance. Similarity matrices for each layer of omic data (**K_u_**, **K_m_**, **K_ucd_**, and **K_ud_**) were calculated as specified in Equation 3 where $\sigma_{u}^{2}$, $\sigma_{m}^{2}$, $\sigma_{\mathrm{uc}}^{2}$, $\sigma_{\mathrm{ud}}^{2}$ were variance parameters associated with GE, MET, and expression profiles under two levels of water availability. Additionally, the model was expanded by the interaction between gene expression and DNA methylation (MET × GE) $um_{ij}∼\mathrm{MVN}\left(0,K_{\mathrm{um}}\sigma_{\mathrm{um}}^{2}\right)$, with **K_um_** = **K_u_** # **K_m_** (# represents cell‐by‐cell multiplication operator, known as the Hadamard product) and $\sigma_{\mathrm{um}}^{2}$ was the variance parameter associated with the interaction term. All variance parameters of the random effects were assigned priors with scaled‐inverted χ^2^ distribution and degrees of freedom close to zero (df0 = 0.0001) and *R* ^2^ parameter set equal to .8, with the default choices implemented using the Bayesian ridge regression specification in the Bayesian generalized linear regression (BGLR) R package.

Apart from aboveground phenotypes, a root trait, DRL, was analyzed in this study. Because there was one root record for each minirhizotron tube (therefore also family), a different model needed to be applied for this trait. The following full model is applied for DRL in Equation 6:

(6) $$y_{ij}=s_{i}+g_{i}+u_{ij}+m_{ij}+um_{ij}+\varepsilon_{ij}$$

where each element was specified as above.

### 2.6 Assessment of predictive ability

The performance of models was evaluated using the leave‐one‐family‐out cross‐validation approach, dividing the data set into n training‐testing subsets (*n* = number of families = 120), which led to 120 combinations. This procedure was repeated 20 times, and a total of 2,400 runs were performed for each training–testing combination. Predictive abilities were calculated as the Pearson's correlation between the families' phenotypes, corrected for the treatment (‘fixed’) effects estimates, and the predicted values for each treatment separately. Family and spatial effects were not included in the calculation of predictive abilities because of the assumption of independence between lines and location within the semifield. Hence, the baseline model was not included in the cross‐validation scheme. Models were compared based on the average Pearson's correlation and standard deviation from each run. The predictive abilities of the models were assessed based on the ability of each model to predict phenotypic traits (above‐ and belowground) after accounting for the general effects of the water availability, that is, the treatment effects. The prediction's degree of inflation or deflation (bias) was measured by estimating the regression coefficient between observed and predicted values. Models with no inflation are expected to have a regression coefficient equal to one. The model fit was also evaluated by the deviance information criterion (DIC) (Spiegelhalter et al., 2002), where a smaller DIC value is considered better.

To evaluate the difference in predictive ability between models for each treatment, the Hotelling–Williams *t* test was applied (Dunn OJ & V, 1971). Differences were considered significant if the *p* value of the test was <.01.

### 2.7 Software

Models were fitted using the R package BGLR (Pérez & de los Campos, 2014) in the R v3.5.1 environment (R Core Team, 2018). For each model, a single chain of 150,000 iterations was run with a burn‐in setting of 20,000, and the remaining samples were thinned at the interval of 20 (Supplemental Figure S3). The convergence of the posterior chains to a stationary state was evaluated following Gelman's and Rubin's approach using chains in the R package CODA (Plummer et al., 2006). Scripts showing how to perform all the analyses are contained in Supplemental File S1.

## 3 RESULTS

### 3.1 Phenotypic data

Descriptive statistics of the four traits analyzed in this study are presented in Table 2. Mean values of traits under drought and control conditions were compared using ANOVA test. The phenotypic range was widest for DRL and narrowest for CEL. There was a statistically significant difference between the two treatments for DW and DMDig but not for CEL on a population level.

**TABLE 2:** Descriptive statistics for dry weight, cellulose content, and dry matter digestibility

Trait	Treatment	No.	Average (SD)	Range	Coefficient of variation
					%
DW (g)	control	120	78.0 (15.8)	40.4–114.7	20.2
	drought	120	100.8 (23.3)	49.7–165.0	23.1
DMDig (percentage of DM)	control	120	89.6 (1.6)	82.1–93.9	1.8
	drought	120	89.0 (1.3)	84.8–92.4	1.5
CEL (percentage of DM)	control	120	21.0 (1.2)	18.3–25.3	5.8
	drought	120	21.0 (1.2)	18.4–25.2	5.8
DRL (cm)	N/A	117	96.4 (43.5)	10.6–225.0	45.2

*Note*. CEL, cellulose content as percentage of DM; DMDig, dry matter digestibility as percentage of DM; DRL, deep root length (one measurement per F_2_ family); DW, herbage dry matter (DM) weight.

### 3.2 Spatial distribution characteristics

**FIGURE 2:**
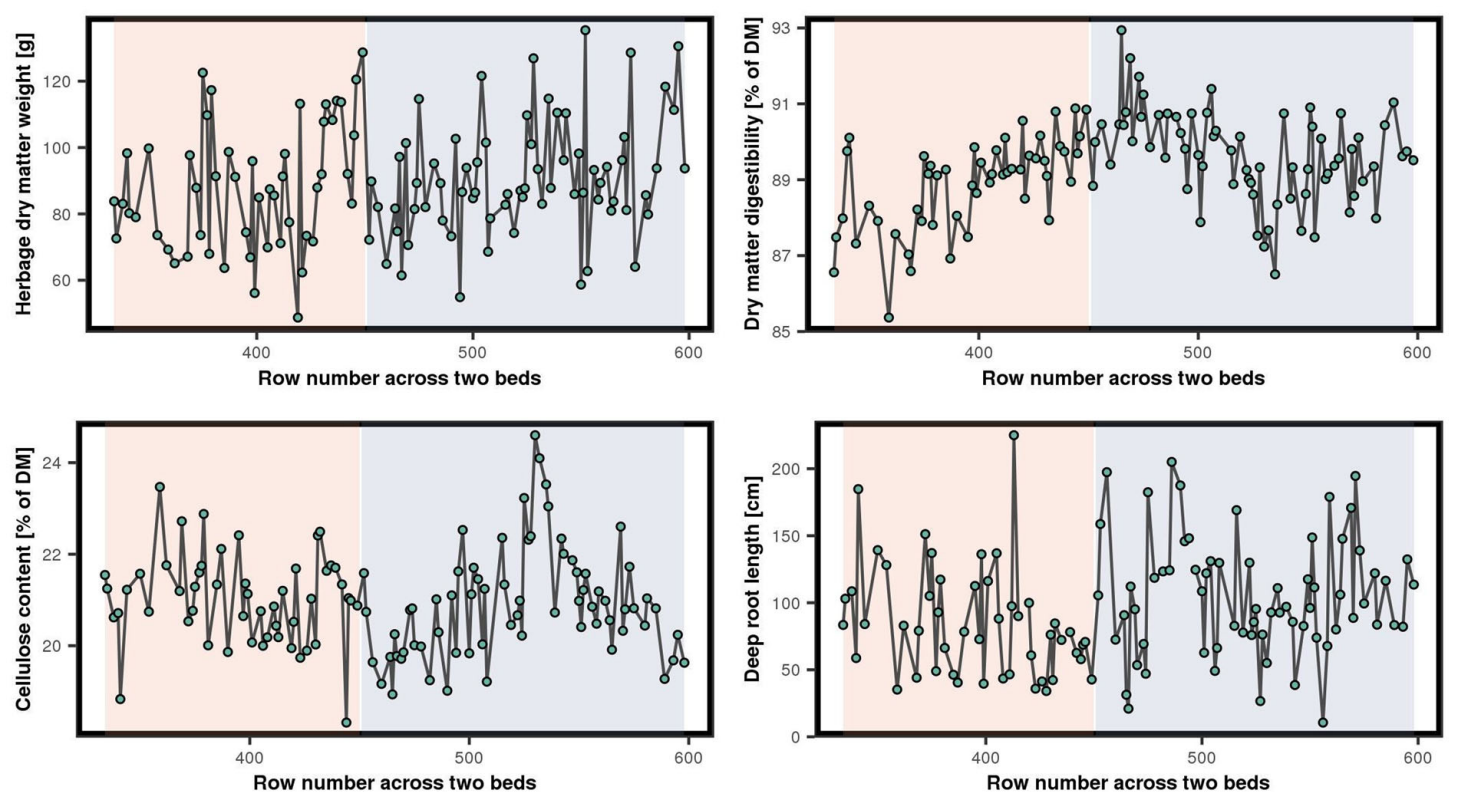
Variation of mean phenotypic values of four analyzed traits across two experimental units (in Beds 3 and 4) in the RadiMax facility. Light orange and light purple backgrounds indicate Bed 3 and Bed 4, respectively. Each row number indicates the position of a unique F_2_ family in the facility

Moran's spatial autocorrelation values were statistically significant (*p* = .001 for all traits), indicating that phenotypic values in the dataset were more spatially clustered than would be expected if underlying spatial processes were random, confirming the need for spatial correction in the predictive models. The variation in phenotypic traits is shown in Figure 2. Spatial patterns across both units of each trait are not a consequence of water availability but nonhomogeneous variation under semifield conditions, for example, uneven soil packing independent of treatment.

### 3.3 Estimates of variance components

Three aboveground traits, DW, CEL, DMDig, and one belowground trait, DRL, were used as response variables in this study. We fitted a sequence of models designed to partition phenotypic variance into its components and evaluate how much more of it can be captured by introducing different layers of omics data. Estimates of variance components derived from the analysis of the aboveground traits are shown in Table 3.

**TABLE 3:** Estimated variance components (95% highest density interval of posterior distributions) derived from Gaussian Bayesian ridge regression model for aboveground traits

Model^a^	$\sigma_{s}^{2}$	$\sigma_{l}^{2}$	$\sigma_{g}^{2}$	$\sigma_{m}^{2}$	$\sigma_{u}^{2}$	$\sigma_{\mathrm{um}}^{2}$	$\sigma_{\mathrm{uc}}^{2}$	$\sigma_{\mathrm{ud}}^{2}$	$\sigma_{\varepsilon }^{2}$	DIC
**Dry matter weight**										
M1	0.17 (0.08–0.26)	0.33 (0.22–0.44)	–	–	–	–	–	–	0.28 (0.21–0.35)	454.8
M2	0.16 (0.07–0.25)	0.17 (0.07–0.27)	0.25 (0.11–0.39)	–	–	–	–	–	0.27 (0.21–0.34)	449.9
M3	0.16 (0.07–0.25)	0.17 (0.07–0.28)	0.24 (0.10–0.39)	0.11 (0.06–0.18)	–	–	–	–	0.17 (0.10–0.25)	384.4
M4	0.13 (0.06–0.21)	0.14 (0.06–0.24)	0.20 (0.07–0.33)		0.21 (0.08–0.35)	–	–	–	0.16 (0.08–0.23)	360.4
M5	0.13 (0.05–0.22)	0.14 (0.05–0.25)	0.20 (0.07–0.34)	0.09 (0.04–0.14)	0.17 (0.06–0.30)	–	–	–	0.11 (0.05–0.18)	300.4
M6	0.13 (0.05–0.22)	0.14 (0.05–0.25)	0.20 (0.06–0.34)	0.07 (0.04–0.12)	0.14 (0.05–0.26)	0.07 (0.03–0.11)	–	–	0.09 (0.03–0.16)	263.3
M7	0.10 (0.03–0.17)	0.09 (0.03–0.16)	0.12 (0.04–0.23)	0.07 (0.03–0.11)	0.12 (0.04–0.22)	0.06 (0.03–0.10)	0.13 (0.04–0.25)	0.09 (0.03–0.18)	0.10 (0.04–0.16)	271.0
**Dry matter digestibility**										
M1	0.28 (0.17–0.41)	0.23 (0.14–0.33)	–	–	–	–	–	–	0.32 (0.24–0.40)	477.6
M2	0.27 (0.15–0.40)	0.13 (0.05–0.21)	0.19 (0.08–0.30)	–	–	–	–	–	0.30 (0.23–0.38)	472.0
M3	0.28 (0.16–0.42)	0.13 (0.05–0.22)	0.20 (0.08–0.33)	0.18 (0.10–0.25)	–	–	–	–	0.11 (0.04–0.19)	277.6
M4	0.21 (0.09–0.34)	0.11 (0.04–0.19)	0.15 (0.06–0.26)	–	0.31 (0.17–0.48)	–	–	–	0.11 (0.05–0.18)	289.0
M5	0.21 (0.09–0.34)	0.11 (0.04–0.19)	0.15 (0.06–0.27)	0.10 (0.04–0.16)	0.20 (0.08–0.34)	–	–	–	0.08 (0.03–0.14)	220.7
M6	0.21 (0.09–0.34)	0.10 (0.03–0.18)	0.15 (0.05–0.26)	0.08 (0.03–0.13)	0.17 (0.06–0.29)	0.08 (0.03–0.12)	–	–	0.06 (0.02–0.11)	174.4
M7	0.12 (0.03–0.22)	0.07 (0.02–0.12)	0.09 (0.03–0.17)	0.07 (0.03–0.13)	0.13 (0.04–0.24)	0.07 (0.03–0.12)	0.12 (0.03–0.23)	0.10 (0.03–0.20)	0.06 (0.02–0.11)	179.4
**Cellulose content**										
M1	0.32 (0.17–0.46)	0.36 (0.24–0.48)	–	–	–	–	–	–	0.25 (0.19–0.32)	437.5
M2	0.31 (0.17–0.46)	0.17 (0.07–0.28)	0.27 (0.13–0.44)	–	–	–	–	–	0.25 (0.19–0.31)	432.1
M3	0.32 (0.17–0.47)	0.17 (0.06–0.30)	0.27 (0.10–0.43)	0.12 (0.06–0.18)	–	–	–	–	0.14 (0.06–0.20)	333.1
M4	0.20 (0.09–0.31)	0.13 (0.06–0.22)	0.19 (0.07–0.34)	–	0.34 (0.22–0.47)	–	–	–	0.05 (0.02–0.09)	141.4
M5	0.21 (0.10–0.32)	0.13 (0.05–0.22)	0.19 (0.06–0.34)	0.05 (0.03–0.08)	0.29 (0.17–0.42)	–	–	–	0.05 (0.02–0.08)	123.7
M6	0.21 (0.09–0.33)	0.12 (0.04–0.22)	0.18 (0.06–0.33)	0.05 (0.02–0.08)	0.26 (0.14–0.40)	0.05 (0.03–0.08)	–	–	0.05 (0.02–0.08)	117.1
M7	0.16 (0.06–0.28)	0.08 (0.03–0.15)	0.11 (0.03–0.21)	0.04 (0.02–0.07)	0.22 (0.11–0.33)	0.04 (0.02–0.07)	0.10 (0.03–0.20)	0.11 (0.03–0.22)	0.05 (0.02–0.08)	123.4

*Note*. DIC, deviance information criterion; $\sigma_{s}^{2}$, spatial neighborhood variance, $\sigma_{l}^{2}$, family or residual genetic variance; $\sigma_{g}^{2}$, genomic variance; and $\sigma_{m}^{2}$, $\sigma_{u}^{2}$, $\sigma_{\mathrm{um}}^{2}$, $\sigma_{\mathrm{uc}}^{2}$, and $\sigma_{\mathrm{ud}}^{2}$ were variance parameters associated with DNA methylation, gene expression, interactions between DNA methylation and gene expression, and expression profiles under two levels of water availability; $\sigma_{\varepsilon }^{2}$, residual variance

^a^ M1–M7 are model numbers of the aboveground traits. The effects included in each of them are described in columns.

The baseline model (M1) included treatment (flat prior), spatial variation, and family capturing between 60 (DMDig) and 73% (CEL) of phenotypic variance $\left(\sigma_{s}^{2}+\sigma_{l}^{2}\right)/\left(\sigma_{s}^{2}+\sigma_{l}^{2}+\sigma_{\varepsilon }^{2}\right)$ (Table 3). Expanding the baseline model by adding genomic effect (M2) had a negligible effect on residual or variance explained by spatial neighborhood. Addition of an additive genomic effect allocated over half of the variance from family to additive genetic effects (M2) for all traits. In both models (M1 and M2), variance captured by spatial effect is moderate for DW (∼20%). However, for two other aboveground traits, variance corresponding to spatial effect is comparable or even bigger than summed family and SNP effect, thus highlighting the importance of accounting for microenvironmental variations under semifield conditions.

When the MET effect was added (M3), the total proportion of variance explained increased by ∼11% for DW and CEL and 22% for DMDig. For all three aboveground traits, the addition of MET contributed almost exclusively to explaining residual variance, which indicates a potential for epigenetic variation to account for a considerable proportion of phenotypic variance of complex traits.

Incorporating the expression profile into the model (M4) further increased the proportion of the variance explained and captured a sizeable proportion of variance resulting from the microenvironment ($\sigma_{s}^{2}$), family, and SNPs. Estimated variance associated with GE varied from 0.21 for DW to 0.31 and 0.34 for DMDig and CEL, respectively. In other words, GE had both genetic and microenvironmental (spatial) components, whereas methylation seemed independent of spatial and genetic structures in the (aboveground) data.

Combining all omics data (M5) and adding the interaction term between MET and GE (M6) did not lead to a substantial reduction in the estimated residual variance (CEL) or did it by only a small margin (from 0.11 to 0.09 for DW and from 0.08 to 0.06 for DMDig) relative to model M4. However, the inclusion of both omics and interaction between the two (M5 and M6) reduced the variance explained by each omics component compared with single omic models (M3 and M4).

Separating the variance explained by the interaction between omics is of interest for understanding how one omic layer is associated with another. Variance estimates from the most comprehensive model (M7) suggest that by the inclusion of various omic layers, as well as interactions between them and treatment, the residual variance may be reduced from 64 (DW) and 80% (CEL and DMDig). A full model (M7) also had the best fit with the lowest DIC for DW and DMDig. Furthermore, the proportion of within‐treatment variation explained by GE × treatment interaction (23–26%) shows the importance of considering such interactions, especially in trials with insufficient randomization or few replicates and significant micro‐ and macroenvironmental variations. However, it is worth noting that parts of the omic and treatment effects could be explained by their interactions in models M6 and M7. Nonetheless, for all three traits, in comparison to M2, incorporation of either MET, GE, or both, led to a substantial increase of phenotypic variance explained by the models, indicating that differences between lines across two treatments cannot be captured alone by marker effect, treatment, and spatial correction. Thus, to some extent, MET and GE contribute to explained phenotypic variance beyond additive genetic profile or microenvironmental condition.

Estimates of variance components for DRL are presented in Table 4. For each minirhizotron tube, there was one record per family available. Hence, models did not include the effect of treatment or family.

**TABLE 4:** Estimated variance components (95% highest density interval of posterior distributions) derived from Gaussian Bayesian ridge regression model for deep root length (root length below 110 cm)

Model^a^	$\sigma_{s}^{2}$	$\sigma_{g}^{2}$	$\sigma_{m}^{2}$	$\sigma_{u}^{2}$	$\sigma_{\mathrm{um}}^{2}$	$\sigma_{\varepsilon }^{2}$	DIC
M1‐R	0.27 (0.13–0.42)	0.60 (0.30–0.93)	–	–	–	0.31 (0.07–0.56)	248.2
M2‐R	0.21 (0.09–0.35)	0.47 (0.19–0.75)	0.26 (0.11–0.44)	–	–	0.23 (0.04–0.45)	213.1
M3‐R	0.21 (0.08–0.34)	0.33 (0.13–0.59)	–	0.37 (0.14–0.63)	–	0.21 (0.05–0.41)	209.7
M4‐R	0.18 (0.06–0.31)	0.28 (0.09–0.51)	0.20 (0.07–0.35)	0.28 (0.10–0.51)	–	0.18 (0.04–0.36)	192.5
M5‐R	0.16 (0.05–0.29)	0.24 (0.07–0.45)	0.17 (0.05–0.33)	0.24 (0.07–0.44)	0.15 (0.05–0.27)	0.17 (0.04–0.35)	188.0

*Note*. DIC: deviance information criterion; $\sigma_{s}^{2}$, spatial neighborhood variance; $\sigma_{g}^{2}$ genomic variance, and $\sigma_{m}^{2}$, $\sigma_{u}^{2}$, and $\sigma_{\mathrm{um}}^{2}$ were variance parameters associated with DNA methylation, gene expression, the interaction between DNA methylation and gene expression; $\sigma_{\varepsilon }^{2}$, residual variance.

^a^ M1‐R–M7‐R are model numbers.

The spatial effect seemed to contribute substantially to variance the amount of variance explained for all five models for DRL to a greater extent than for aboveground traits. In the baseline model (M1‐R), for root phenotype, additive genetic variance ($\sigma_{g}^{2}$) accounted for 0.60 of the total variance, over double of the spatial effect (Table 4). The introduction of two single omics (M2‐R and M3‐R) contributed to the total variance explained and captured a significant proportion of spatial and genotypic variation. In contrast to aboveground traits, the variance of DRL captured by MET (M3‐R) was similar to SNP and GE, contributing equally to reducing the residual variance. Finally, combining all three omics data and interaction of MET and GE in M4‐R and M5‐R incrementally reduced residual variance down to 0.17 and affected variance components values for spatial and main effects of markers. A full model (M5‐R) also showed the best fit with the lowest DIC value (188.0) out of all five models.

### 3.4 Assessment of predictive ability

**FIGURE 3:**
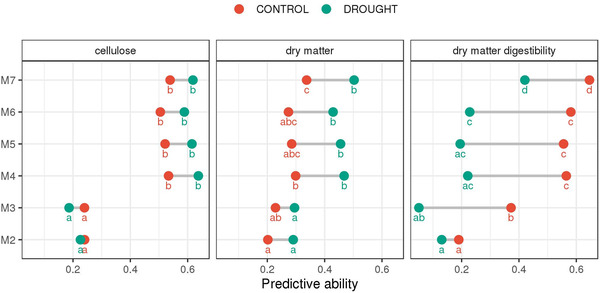
Leave‐one‐family‐out cross‐validations for all models and aboveground traits. Predictive ability is expressed as Pearson's correlation between predicted and observed values for control and drought. Different letters indicate significant (*p* ≤ .01) differences between models within each treatment and trait. Baseline model (M1) is not included in the figure as family, and spatial effects were not included in the calculation of predictive abilities

Leave‐one‐family‐out cross‐validation scheme was used to assess each model's ability to predict phenotypic traits of families (under both treatments) not used to train the model. For each family, models were fitted using data from the rest of the analyzed population, and the scheme was repeated 25 times, resulting in 25 predicted values for each family. Figure 3 shows the average Pearson's correlation between predicted and observed values of aboveground traits for each model analyzed and obtained for each treatment separately. Model M2 yielded the lowest prediction correlations: between 0.13 and 0.19 for DMDig and 0.20 and 0.29 for DW and CEL. On the other hand, the model extended by MET (M3) achieved similarly moderate prediction correlations with the highest value for DMDig under control at 0.39. Including GE (M4–M6) doubled predictive abilities in comparison to M2 and M3 for CEL (0.53–0.63) and substantially increased for DMDig (up to 0.59 for control and 0.25 for drought) and DW under drought (0.43–0.46). The full model (M7) achieved the highest correlation values between predicted and observed aboveground phenotypes. However, for CEL under drought (M4–M7) and DMDig under control (M4–M7), the overestimation of prediction seemed a potential issue for models as presented in Supplemental Figure S4. There was no significant inflation in the predicted values for the rest models and traits (including DRL and DW).

**FIGURE 4:**
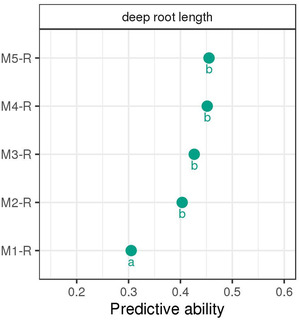
Leave‐one‐family‐out cross‐validations for deep root length. Predictive ability is expressed as Pearson's correlation between predicted and observed values. Different letters indicate significant (*p* ≤ .01) differences between models

The predictive abilities of DRL are presented in Figure 4. The addition of MET and GE significantly increased predictive ability for the root length phenotype compared with M1‐R. Compared with the aboveground phenotypes (Figure 3), MET also contributed significantly to the increase of models' predictive ability. In general, there was little difference between the full model and M3 and M4, with prediction oscillating around 0.45. The test for variance inflation did not indicate a significant under‐ or overdispersion in DRL predictions (Supplemental Figure S5).

## 4 DISCUSSION

In recent years, the availability of different types of omic data has increased because of reduced sequencing costs, the development of public databases, and increased computing power. These data sets are large, multilayered, and highly dimensional. Although the methodology is available for analyzing individual data sets, there is still a lack of consensus on integrating multiple layers of such data to better understand the basis and relative effects of the omic layers on complex traits in crops.

In this study, we investigated the relative contributions of different omic predictors to partition variance components for a better understanding of the different omics layers' effects on four traits related to yield, quality, and DRL in a semifield state‐of‐the‐art root phenotyping facility. The plants were exposed to two different water treatments: drought and control (Svane, Dam, et al., 2019) (Figure 1).

We implemented a series of Bayesian regression models (Table 1) with a Gaussian prior to accommodate heterogeneous, high‐dimensional omics inputs, account for nonlinear interactions between and within layers, omic × treatment (drought) interactions, and handling quantitative traits. By analyzing the before‐mentioned phenotypes (Table 2) with different omics layers and accounting for interactions and spatial structures, we could quantify the effects of genomic, MET, and transcriptomic data on the traits analyzed (Tables 3 and 4). Additionally, extending the model beyond SNPs showed a decrease in residual variance. Although genetic markers can also model nonadditive genetic effects (epistasis, dominance), the power to explain variance is limited and dependent on the relationship between individuals. Considering the population of 120 F_2_ families (genotyped on a family basis), a comparison of marker‐ and omics‐based nonadditive genetic effects was not feasible.

Gene expression accounted for considerable parts of spatial and additive genetic variance for all phenotypes, but after partitioning out spatial, additive genetic, and GE × treatment effects, a large portion of variance was still explained by GE. This probably could be explained by the fact that GE provides a link between the analyzed trait and variation that cannot be explained by DNA‐based genetic markers. In contrast, MET mostly accounted for a sizeable proportion of the residual variance for the quality traits but, interestingly, captured larger proportions of spatial and additive genetic variance for the deep root length.

Experimental and agricultural fields are rarely homogeneous, exhibiting variability in landscape attributes, soil properties, water, or nutrient availability. Effects on analyzed phenotypic traits can stem from soil compactness, nutrient availability, or even thermal distribution in the semifield. Spatial variation of the RadiMax facility was shown based on Beds 1 and 2 (Guo et al., 2020). In our data, we identified that phenotypic traits of perennial ryegrass grown in Beds 3 and 4 also were spatially clustered (Figure 2), confirming the need to account for a nonrandom distribution of spatial effects in the semifield facility. The spatial effect was relatively high for all models (Tables 3 and 4). Together with the treatment effect in the model, the introduction of the spatial effect explained micro‐ and macroenvironment in the semifield in a more comprehensive way. Furthermore, accounting for spatial effects allowed for better variance partitioning, highlighting nonadditive genetic effects and regulatory effects independent of the additive genetic architecture of the omics components in the models. Accounting for the spatial effects can be an effective means to reduce the noise level in nonuniform data, as shown here and by Guo et al. (2020), and can be particularly useful for datasets with limited numbers of trials. Spatial effects can also account for otherwise unexplained noise in randomized experiments with many replicates.

Because the genetic effect on the phenotype is passed from the genome through multiple regulatory layers like MET patterns and GE, a combination of these downstream regulatory layers can compensate for incomplete information in any single regulatory layer and provide a more thorough investigation of genotype–phenotype associations (Ritchie et al., 2015). However, it is important to remember that, unlike DNA sequence information, methylation patterns and transcriptome information are not stably inherited in the population. Their levels are affected by environmental conditions and factors like plant tissue, conditions at sampling, and environmental effects on the plant until sampling. Nevertheless, GE (along with GE_C_ and GE_D_) consistently explained the highest proportion of variance captured by models compared with MET and SNP for the aboveground traits and showed excellent predictive ability in models M4–M7 (Figure 3). Similar results regarding improved phenotype prediction of complex traits were shown before (Azodi et al., 2020; Fu et al., 2012; Westhues et al., 2017). Gene expression explained a substantial part of the genetic variance for all the investigated phenotypes, and models with GE had higher prediction accuracy than models without (Table 3). The model with MET as the single omic was better for predicting root intensity than the model with GE or a combination of GE and MET (Figure 4). DNA methylomic explained residual variance not accounted for by SNPs or GE (Table 3) for aboveground traits but only for DMDig was this reflected in increased predictive ability.

Changes in epigenetic (MET) patterns likely act slower than fluctuations in GE. Variability in MET profiles between plants can be caused by technical noise, developmental stochasticity, and microenvironmental variance, as well as being coupled with genetic variation (Banta & Richards, 2018; Richards, 2006). However, epigenetic diversity is also expected to be a consequence of transgenerational epigenetic variation, which is primarily a nongenetic phenomenon (Richards, 2006) that can influence qualitative and quantitative traits (Banta & Richards, 2018; Noshay & Springer, 2021; Varona et al., 2015). One aim of our multiomic prediction models was to partition transcriptomic and epigenetic variance from the total genetic variance. Thus, there is a need to identify and account for nongenetic variance in the model evaluation and prediction.

In contrast to GE, MET was not confounded by spatial effects for the aboveground traits (Table 3), whereas the opposite was true for DRL (Table 4). This may be because the aboveground phenotypes were obtained from the third cut, meaning the regrowth after the second cut. Although cutting also affects root growth and biomass (Vinther, 2006), that is, frequent cutting reduces both traits, it is reasonable to assume that the aboveground phenotypes represented a shorter growth window than the root trait.

Additionally, changes in the microenvironment may happen faster aboveground because of within‐field differences in shading during the day, exposure to wind, or relative humidity on top of soil heterogeneity, which is more persistent. These rapid changes might affect the aboveground phenotypes more than the roots. Gene expression effects captured a spatial component for both aboveground and root traits. Longer lasting spatial effects are more likely to induce changes in MET patterns. Because the spatial component of MET for DRL was considerable, we speculate that factors contributing to soil heterogeneity, including water and nutrient availability, triggered changes in MET. This also indicates a potentially important role of MET in shaping perennial ryegrass phenotypes during a growing season as a response to the microenvironment. Cortijo et al. (2014) suggested an important role of methylation on primary root length in *Arabidopsis*. Further studies are needed to identify to what extent heritable, differentially methylated sites or regions affect phenotypes and can be used in plant breeding.

Adding both GE and MET to the models for aboveground traits did not increase predictive ability compared with models with only GE (Figure 3). However, these models usually had a better DIC fit, perhaps because of more precise variance allocation, which reduces bias (Ni et al., 2019). The GE × MET interaction term was usually minor compared with the variance explained by each of the omics in the model but relatively larger for DRL, indicating a sizeable modifying effect of one omic on the other. González‐Reymúndez et al. (2017) suggested that larger sample sizes may be needed to get the full benefits of including omic × omic interactions in prediction models.

Although the spatial effects explained the by‐row heterogeneity, the different ryegrass families were also expected to have a heterogeneous response to the different water treatments. Therefore, including the GE × treatment interaction term in the model (M7) for the aboveground traits could identify the magnitude of the phenotypic variance depending on the treatment (Table 3). Thus, the GE × treatment interaction explained more residual variance than the simpler models and increased predictive ability (Figure 3). Likewise, the interaction terms separated variance caused by treatment from the remaining genetic component of the GE matrix, resulting in a more precisely allocated variance.

Our analysis allowed us to consider multiple variables simultaneously (from environmental to multiomics components), which resulted in enhanced predictive abilities of more complex models. Overall, integration of additional predictors can help dissect the complex traits and further strengthen models' predictive abilities. However, in our cross‐validation scheme, we observed heterogeneity in predictive abilities under different treatments and a degree of inflation of some of the predicted values for DMDig and CEL. Depending on the treatment for which it was explored, differences between predictive ability values could be due to the sample size of the analyzed population, which did not allow for the exploration of enough phenotypic heterogeneity to achieve a desired degree of generalizability. It could also be speculated that differential treatment effect, which was mainly pronounced on the level of GE and MET patterns compared with phenotypic traits (Table 2), lies at the source of observed discrepancies in predictive abilities under control and drought.

We have partitioned variance components to better understand the effect of three omic layers and showed the importance of accounting for the heterogeneity of experimental semifields. Both MET and GE play important roles in shaping phenotypes, which was illustrated by the variance explained by these predictors (Tables 3 and 4). An alternative hypothesis of complex trait causation states that phenotypic variation is not hierarchical but an interconnection between variations across omic levels. (Ritchie et al., 2015) By combining multiple omics layers, we assume that we capture a larger part of these interconnections and bridge the genotype–phenotype gap. In our study, we partitioned the variance to identify the relative effects of each layer on the phenotypes and how the different regulatory layers were connected in terms of unique and overlapping variance.

The partitioning also quantified additive and more complex components of the variance explained by MET and GE. Recently, important steps have been taken to obtain breeding values using an intermediate omic feature in simulated data sets (Christensen et al., 2021; Weishaar et al., 2020). Importantly, any increase in prediction accuracy of breeding values would depend on the relationship between the omics features and the phenotype and heritability of omics features because only the genetic part of each omics feature is passed on to the next generation. However, validating the methods on real data is not straightforward, as naïve cross‐validation may favor methods that predict accurate phenotypes and not accurate breeding values (Christensen et al., 2021). Although much work is needed to develop the methods and validate their suitability on real data, advances like this show that multiomic data can successfully improve future breeding value estimation. This is supported by our findings that there are unique and sizeable genetic components of both MET and GE data, which is a prerequisite for improving the accuracy.

The data set comprised a unique combination of transcriptomic, methylomic, and genetic data, as well as relevant phenotypes, which made it suitable for the development and testing of the models and assessment of variance components. The model framework can be applied to any multiomic dataset to reveal the relative importance of the variance components. Compared with the data set dimensions, the study's main limitation is a relatively small sample size. Further research with a larger sample size would allow extending the scope of analysis to include epistatic and dominance effects as well as to improve the prediction accuracy, eliminate possible bias, and strengthen the conclusion. Nevertheless, this study presents a partitioning of the phenotypic variance into different regulatory layers for the first time. This will pave the way for future studies as the cost of obtaining multiomics data will continue to decrease.

## AUTHOR CONTRIBUTIONS

Marta Malinowska: Conceptualization; Formal analysis; Investigation; Methodology; Writing – original draft. Anja Karine Ruud: Conceptualization; Formal analysis; Investigation; Writing – original draft. Just Jensen: Methodology; Supervision; Writing – review & editing. Simon Fiil Svane: Data curation; Investigation; Writing – review & editing. Abraham George Smith: Data curation; Investigation; Writing – review & editing. Andrea Bellucci: Data curation; Writing – review & editing. Ingo Lenk: Data curation; Writing – review & editing. Istvan Nagy: Data curation; Writing – review & editing. Mattia Fois: Investigation; Writing – review & editing. Thomas Didion: Data curation; Writing – review & editing. Kristian Thorup‐Kristensen: Funding acquisition; Project administration; Resources; Supervision. Christian Sig Jensen: Funding acquisition; Project administration; Resources. Torben Asp: Funding acquisition; Project administration; Resources; Supervision; Writing – review & editing.

## CONFLICT OF INTEREST

The authors declare no conflict of interest. The founding sponsors had no role in the design of the study nor in the collection, analysis, or interpretation of data and the writing of the manuscript.

## Supporting information

Supplemental Figure S1. Volumetric water content (VWC) measurements between April and August across two beds. The subplots to (a) represent the mean VWC of three soil layers in the middle of each bed represented by different colors with the corresponding standard error shown as a grey shade (*n*_50cm_ = 8, *n*_100cm_ = 4, *n*_150cm_ = 4). Field capacity (FC) and permanent wilting point (PWP) are shown as dashed blue and red lines, respectively. The subplot (b) show the mean VWC data where solid lines with different colors represent the mean soil water content at nine locations with sensors installed 25 cm above the sub‐surface irrigation system (*n* = 4). The grey shade represents the minimum and maximum water content measured by all 36 sensors. Field capacity (FC) and permanent wilting point (PWP) are shown as dashed blue and red lines, respectively
Supplemental Figure S2 Correlation plot between segmented skeleton length (*x*‐axis) from the CNN and manual grid count (y‐axis)
Supplemental Figure S3. Combined trace plots showing the distribution of the values of each parameter in the chain for the full model predicting A) DW; B) DMDig; C) CEL, and D) DRL
Supplemental Figure S4. Inflation of prediction for the aboveground trait. The *y*‑axis is the regression coefficient of predicted on observed values; the *x*‑axis is the model number. Horizontal line is regression coefficient of 1. In a situation of no inflation, the regression coefficient for each model should not be significantly different from 1. Based on a *t*‐test, ‘NS’ (not significant) and ‘*’ (p < 0.01) indicate whether coefficient is significantly different from 1 or not.
Supplemental Figure S5. Inflation of prediction for the deep root length. The y‑axis is the regression coefficient of predicted on observed values; the x‑axis is the model number. Horizontal line is regression coefficient of 1. In a situation of no inflation, regression coefficient for each model should not be significantly different from 1. Based on a *t*‐test, ‘NS’ (not significant) and ‘*’ (p < 0.01) indicate whether coefficient is significantly different from 1 or not.

Supplemental File S1. R Scripts

Supplementary File S2. RData file with the input data described in Supplementary File S1

## Data Availability

All relevant data can be found within the manuscript and its Supplemental Materials.
