# Supplementary material for: Relative importance of genotype, gene expression, and DNA methylation on complex traits in perennial ryegrass
Source: Plant Genome. 2022 Aug 17;15(4):e20253. doi: 10.1002/tpg2.20253 (PMC12807231; doi:10.1002/tpg2.20253)
Supplement: Supplementary file 1 [file TPG2-15-e20253-s001.docx]

The following document illustrates how the analysis presented in the manuscript was performed.

**Installing the packages:**

```
# Install BGLR and bayestestR packages
#install.packages("BGLR")
#install.packages("bayestestR")
```

**Loading data:**

The TPG_DATA.RData file contains the following objects

- X.ge: a similarity matrix for gene expression.
- X.geC a similarity matrix for gene expression under “wet”
- X.geD a similarity matrix for gene expression under “dry”
- X.meth: a similarity matrix for methylation values.
- X.mXg an interaction matrix between methylation and gene expression data
- G: a Gmatrix for SNP genotypes
- spat: spatial distance weights obtained from the spatial connectivity matrix
- XTRT: incidence matrix for treatment
- Xfam incidence matrix for family
- y: a matrix with id and phenotypic trait, Phenotypic data is centered and scaled

load('TPG_DATA.RData')

ls()

\# "G" "spat" "y" "X.ge" "X.geC" "X.geD" "X.met" "X.mXg" "Xfam" "XTRT"

**Obtaining principal components**

The following code shows how to obtain the PC for the SNP data. Same approach can be applied to other data used in the model:

```
EVD<-eigen(G,symmetric=TRUE)
tmp <- EVD$values >  1e-07
pc.snp <- EVD$vectors[,tmp] %*% diag(sqrt(EVD$values[tmp]))
rownames(pc.snp) <- rownames(G)
colnames(pc.snp) <- paste0("PC",1:ncol(pc.snp))

# #Correct eigenvalue for the effect of line (120 lines vs 240 observations)
y$short_id<-factor(x=y$short_id,levels=rownames(G),ordered=TRUE)
id<-model.matrix(~y$short_id-1)
ID<-(y$ID)
rownames(id) <- ID

#Correct eigenvalue for the effect of line (120 lines vs 240 observations)(only to be done for pc.snp and spat
pc.snp<-id%*%pc.snp
dim(pc.snp)
```

**Models’ definition**

nIter=150000 #number of iteriations

burnIn=20000 #burn in

thin=20

verbose=F

**Preparing predictors**

```
ETA=list(
    trt=list(X=XTRT,model='FIXED'),
    spat=list(X=pc.spat,model='BRR',saveEffects=T),
    family=list(X=Xfam,model='BRR',saveEffects=T),
    SNP=list(X=pc.snp,model='BRR',saveEffects=T),
    MET=list(X=pc.met,model='BRR',saveEffects=T),
    GE=list(X=pc.ge,model='BRR',saveEffects=T),
    GExC=list(X=pc.geXc,model='BRR',saveEffects=T),
    GExD=list(X=pc.geXd,model='BRR',saveEffects=T),
    METxGE=list(X=pc.mXg,model='BRR',saveEffects=T))

## ANOVA-Table
mod<-c('M1','M2','M3','M4','M5','M6','M7')
tr<-c('spat','family','snp','met','ge','mXg','geXc','geXd','residual','DIC')
VAR=matrix(nrow=length(mod),ncol=length(tr),NA)
colnames(VAR)=tr
rownames(VAR)=mod

#Highest posterior density region table
tr<-c('spat.low', 'spat.high', 'family.low', 'family.high', 'snp.low', 'snp.high', 'met.low', 'met.high', 'ge.low', 'ge.high', 'mXg.low', 'mXg.high', 'geXc.low', 'geXc.high', 'geXd.low', 'geXd.high', 'residual.low', 'residual.high')
HPD=matrix(nrow=length(mod),ncol=length(tr),NA)
colnames(HPD)=tr
rownames(HPD)=mod
```

**Fitting models**

```
# Fitting baseline model
fm.M1 = BGLR(y = y[,”trait”], ETA = ETA[c('trt','spat','family')], nIter = nIter, burnIn = burnIn, thin=thin, verbose = verbose, saveAt = ‘M1_', df0 = 0.0001, R2 = 0.8)
save(fm.M1,file='fm.M1.RData')

#retriving variance components and Highest Density Interval of posterior distributions
VAR['M1','DIC']=fm.M1$fit$DIC
varE=scan("M1_varE.dat")
VAR['M1','residual']= fm.M1$varE

HPD['M1','residual.low']=hdi(varE, ci=0.95)[,2]
HPD['M1','residual.high']=hdi(varE, ci=0.95)[,3]

B.SP=readBinMat('M1_ETA_spat_b.bin')
V.SP=apply(FUN=var,X=(tcrossprod(pc.spat,B.SP)),MARGIN=2)

VAR['M1','spat']=mean(V.SP)
HPD['M1','spat.low']=hdi(V.SP, ci=0.95)[,2]
HPD['M1','spat.high']=hdi(V.SP, ci=0.95)[,3]

B.FAM=readBinMat('M1_ETA_family_b.bin')
V.FAM<-apply(FUN=var,X=(tcrossprod(XFAM,B.FAM)),MARGIN=2)
VAR['M1','family']=mean(V.FAM)
HPD['M1','family.low']=hdi(V.FAM, ci=0.95)[,2]
HPD['M1','family.high']=hdi(V.FAM, ci=0.95)[,3]
```

#### #Fitting a model for fixed effect, random line and spatial effect, and genomic information

```
fm.M2 = BGLR(y = y[,”trait”], ETA = ETA[c('trt', 'spat', 'family', 'SNP')], nIter = nIter, burnIn = burnIn, thin = thin, verbose = verbose, saveAt = 'M2_', df0 = 0.0001, R2 = 0.8)
save(fm.M2,file = 'fm.M2.RData')

#retrieving variance components and Highest Density Interval of posterior distributions
VAR['M2','DIC']=fm.M2$fit$DIC
varE=scan("M2_varE.dat")
VAR['M2','residual']= fm.M2$varE
HPD['M2','residual.low']=hdi(varE, ci=0.95)[,2]
HPD['M2','residual.high']=hdi(varE, ci=0.95)[,3]

B.SP=readBinMat('M2_ETA_spat_b.bin')
V.SP=apply(FUN=var,X=(tcrossprod(pc.spat,B.SP)),MARGIN=2)
VAR['M2','spat']=mean(V.SP)
HPD['M2','spat.low']=hdi(V.SP, ci=0.95)[,2]
HPD['M2','spat.high']=hdi(V.SP, ci=0.95)[,3]

B.FAM=readBinMat('M2_ETA_family_b.bin')
V.FAM<-apply(FUN=var,X=(tcrossprod(XFAM,B.FAM)),MARGIN=2)
VAR['M2','family']=mean(V.FAM)
HPD['M2','family.low']=hdi(V.FAM, ci=0.95)[,2]
HPD['M2','family.high']=hdi(V.FAM, ci=0.95)[,3]

B.SNP=readBinMat('M2_ETA_SNP_b.bin')
V.SNP<-apply(FUN=var,X=(tcrossprod(pc.snp,B.SNP)),MARGIN=2)
VAR['M2','snp']=mean(V.SNP)
HPD['M2','snp.low']=hdi(V.SNP, ci=0.95)[,2]
HPD['M2','snp.high']=hdi(V.SNP, ci=0.95)[,3]
```

#### # Fitting a model for fixed effect, random line and spatial effects, and 2 omics

```
fm.M3 = BGLR(y = y[,”trait”],ETA=ETA[c('trt', 'spat', 'family', 'SNP', 'MET')], nIter = nIter, burnIn = burnIn, thin = thin, verbose = verbose, saveAt = ‘M3_', df0 = 0.0001, R2 = 0.8)
save(fm.M3,file='fm.M3.RData')

#retrieving variance components and Highest Density Interval of posterior distributions
VAR['M3','DIC']=fm.M3$fit$DIC

varE=scan("M3_varE.dat")
VAR['M3','residual']= fm.M3$varE
HPD['M3','residual.low']=hdi(varE, ci=0.95)[,2]
HPD['M3','residual.high']=hdi(varE, ci=0.95)[,3]

B.SP=readBinMat('M3_ETA_spat_b.bin')
V.SP=apply(FUN=var,X=(tcrossprod(pc.spat,B.SP)),MARGIN=2)
VAR['M3','spat']=mean(V.SP)
HPD['M3','spat.low']=hdi(V.SP, ci=0.95)[,2]
HPD['M3','spat.high']=hdi(V.SP, ci=0.95)[,3]

B.FAM=readBinMat('M3_ETA_family_b.bin')
V.FAM<-apply(FUN=var,X=(tcrossprod(XFAM,B.FAM)),MARGIN=2)
VAR['M3','family']=mean(V.FAM)
HPD['M3','family.low']=hdi(V.FAM, ci=0.95)[,2]
HPD['M3','family.high']=hdi(V.FAM, ci=0.95)[,3]

B.SNP=readBinMat('M3_ETA_SNP_b.bin')
V.SNP<-apply(FUN=var,X=(tcrossprod(pc.snp,B.SNP)),MARGIN=2)
VAR['M3','snp']=mean(V.SNP)
HPD['M3','snp.low']=hdi(V.SNP, ci=0.95)[,2]
HPD['M3','snp.high']=hdi(V.SNP, ci=0.95)[,3]

B.MET=readBinMat('M3_ETA_MET_b.bin')
V.MET=apply(FUN=var,X=(tcrossprod(pc.met,B.MET)),MARGIN=2)
VAR['M3','met']=mean(V.MET)
HPD['M3','met.low']=hdi(V.MET, ci=0.95)[,2]
HPD['M3','met.high']=hdi(V.MET, ci=0.95)[,3]
```

#### # Fitting a model for fixed effect, random line and spatial effects, and 2 omics

```
fm.M4 = BGLR(y = y[,”trait”], ETA = ETA[c('trt', 'spat', 'family', 'SNP', 'GE')], nIter = nIter, burnIn = burnIn, thin = thin, verbose = verbose, saveAt = ‘M4_', df0 = 0.0001, R2 = 0.8)
save(fm.M4,file='fm.M4.RData')

#retrieving variance components and Highest Density Interval of posterior distributions
VAR['M4','DIC']=fm.M4$fit$DIC

varE=scan("M4_varE.dat")
VAR['M4','residual']= fm.M4$varE
HPD['M4','residual.low']=hdi(varE, ci=0.95)[,2]
HPD['M4','residual.high']=hdi(varE, ci=0.95)[,3]

B.SP=readBinMat('M4_ETA_spat_b.bin')
V.SP=apply(FUN=var,X=(tcrossprod(pc.spat,B.SP)),MARGIN=2)
VAR['M4','spat']=mean(V.SP)
HPD['M4','spat.low']=hdi(V.SP, ci=0.95)[,2]
HPD['M4','spat.high']=hdi(V.SP, ci=0.95)[,3]

B.FAM=readBinMat('M4_ETA_family_b.bin')
V.FAM<-apply(FUN=var,X=(tcrossprod(XFAM,B.FAM)),MARGIN=2)
VAR['M4','family']=mean(V.FAM)
HPD['M4','family.low']=hdi(V.FAM, ci=0.95)[,2]
HPD['M4','family.high']=hdi(V.FAM, ci=0.95)[,3]

B.SNP=readBinMat('M4_ETA_SNP_b.bin')
V.SNP<-apply(FUN=var,X=(tcrossprod(pc.snp,B.SNP)),MARGIN=2)
VAR['M4','snp']=mean(V.SNP)
HPD['M4','snp.low']=hdi(V.SNP, ci=0.95)[,2]
HPD['M4','snp.high']=hdi(V.SNP, ci=0.95)[,3]

B.GE=readBinMat('M4_ETA_GE_b.bin')
V.GE=apply(FUN=var,X=(tcrossprod(pc.ge,B.GE)),MARGIN=2)
VAR['M4','ge']=mean(V.GE)
HPD['M4','ge.low']=hdi(V.GE, ci=0.95)[,2]
HPD['M4','ge.high']=hdi(V.GE, ci=0.95)[,3]
round(HPD,2)

#Fitting a model for fixed effect, random line and spatial effects, and 3 omics

fm.M5 = BGLR(y = y[,”trait”], ETA = ETA[c('trt', 'spat', 'family', 'SNP', 'MET', 'GE')], nIter = nIter, burnIn = burnIn, thin = thin, verbose = verbose, saveAt = ‘M5_', df0 = 0.0001, R2 = 0.8)
save(fm.M5,file='fm.M5.RData')

#retrieving variance components and Highest Density Interval of posterior distributions
VAR['M5','DIC']=fm.M5$fit$DIC

varE=scan("M5_varE.dat")
VAR['M5','residual']= fm.M5$varE
HPD['M5','residual.low']=hdi(varE, ci=0.95)[,2]
HPD['M5','residual.high']=hdi(varE, ci=0.95)[,3]

B.SP=readBinMat('M5_ETA_spat_b.bin')
V.SP=apply(FUN=var,X=(tcrossprod(pc.spat,B.SP)),MARGIN=2)
VAR['M5','spat']=mean(V.SP)
HPD['M5','spat.low']=hdi(V.SP, ci=0.95)[,2]
HPD['M5','spat.high']=hdi(V.SP, ci=0.95)[,3]

B.FAM=readBinMat('M5_ETA_family_b.bin')
V.FAM<-apply(FUN=var,X=(tcrossprod(XFAM,B.FAM)),MARGIN=2)
VAR['M5','family']=mean(V.FAM)
HPD['M5','family.low']=hdi(V.FAM, ci=0.95)[,2]
HPD['M5','family.high']=hdi(V.FAM, ci=0.95)[,3]

B.SNP=readBinMat('M5_ETA_SNP_b.bin')
V.SNP<-apply(FUN=var,X=(tcrossprod(pc.snp,B.SNP)),MARGIN=2)
VAR['M5','snp']=mean(V.SNP)
HPD['M5','snp.low']=hdi(V.SNP, ci=0.95)[,2]
HPD['M5','snp.high']=hdi(V.SNP, ci=0.95)[,3]

B.MET=readBinMat('M5_ETA_MET_b.bin')
V.MET=apply(FUN=var,X=(tcrossprod(pc.met,B.MET)),MARGIN=2)
VAR['M5','met']=mean(V.MET)
HPD['M5','met.low']=hdi(V.MET, ci=0.95)[,2]
HPD['M5','met.high']=hdi(V.MET, ci=0.95)[,3]

B.GE=readBinMat('M5_ETA_GE_b.bin')
V.GE=apply(FUN=var,X=(tcrossprod(pc.ge,B.GE)),MARGIN=2)
VAR['M5','ge']=mean(V.GE)
HPD['M5','ge.low']=hdi(V.GE, ci=0.95)[,2]
HPD['M5','ge.high']=hdi(V.GE, ci=0.95)[,3]
```

#### #Fitting a model for fixed effect, random line and spatial effects and 3 omics and their interactions

```
fm.M6 = BGLR(y = y[,”trait”], ETA = ETA[c('trt', 'spat', 'family', 'SNP', 'MET', 'GE', 'METxGE')], nIter = nIter, burnIn = burnIn, thin = thin, verbose = verbose, saveAt = ‘M6_', df0 = 0.0001, R2 = 0.8)
save(fm.M6,file='fm.M6.RData')

#retrieving variance components and Highest Density Interval of posterior distributions
VAR['M6','DIC']=fm.M6$fit$DIC

varE=scan("M6_varE.dat")
VAR['M6','residual']= fm.M6$varE
HPD['M6','residual.low']=hdi(varE, ci=0.95)[,2]
HPD['M6','residual.high']=hdi(varE, ci=0.95)[,3]

B.SP=readBinMat('M6_ETA_spat_b.bin')
V.SP=apply(FUN=var,X=(tcrossprod(pc.spat,B.SP)),MARGIN=2)
VAR['M6','spat']=mean(V.SP)
HPD['M6','spat.low']=hdi(V.SP, ci=0.95)[,2]
HPD['M6','spat.high']=hdi(V.SP, ci=0.95)[,3]

B.FAM=readBinMat('M6_ETA_family_b.bin')
V.FAM<-apply(FUN=var,X=(tcrossprod(XFAM,B.FAM)),MARGIN=2)
VAR['M6','family']=mean(V.FAM)
HPD['M6','family.low']=hdi(V.FAM, ci=0.95)[,2]
HPD['M6','family.high']=hdi(V.FAM, ci=0.95)[,3]

B.SNP=readBinMat('M6_ETA_SNP_b.bin')
V.SNP<-apply(FUN=var,X=(tcrossprod(pc.snp,B.SNP)),MARGIN=2)
VAR['M6','snp']=mean(V.SNP)
HPD['M6','snp.low']=hdi(V.SNP, ci=0.95)[,2]
HPD['M6','snp.high']=hdi(V.SNP, ci=0.95)[,3]

B.MET=readBinMat('M6_ETA_MET_b.bin')
V.MET=apply(FUN=var,X=(tcrossprod(pc.met,B.MET)),MARGIN=2)
VAR['M6','met']=mean(V.MET)
HPD['M6','met.low']=hdi(V.MET, ci=0.95)[,2]
HPD['M6','met.high']=hdi(V.MET, ci=0.95)[,3]

B.GE=readBinMat('M6_ETA_GE_b.bin')
V.GE=apply(FUN=var,X=(tcrossprod(pc.ge,B.GE)),MARGIN=2)
VAR['M6','ge']=mean(V.GE)
HPD['M6','ge.low']=hdi(V.GE, ci=0.95)[,2]
HPD['M6','ge.high']=hdi(V.GE, ci=0.95)[,3]

B.METxGE=readBinMat('M6_ETA_METxGE_b.bin')
V.METxGE=apply(FUN=var,X=(tcrossprod(pc.mXg,B.METxGE)),MARGIN=2)
VAR['M6','mXg']=mean(V.METxGE)
HPD['M6','mXg.low']=hdi(V.METxGE, ci=0.95)[,2]
HPD['M6','mXg.high']=hdi(V.METxGE, ci=0.95)[,3]
```

#### #Fitting a model for fixed effect, random line and spatial effect as well as 3 omics, their interactions and treatment effect on transcriptomic data

```
fm.M7 = BGLR(y = y[,”trait”], ETA = ETA[c('trt', 'spat', 'family', 'SNP', 'MET', 'GE', 'GExC', 'GExD', 'METxGE')], nIter = nIter, burnIn = burnIn, thin = thin, verbose = verbose, saveAt = ‘M7_', df0 = 0.0001, R2 = 0.8)
save(fm.M7,file='fm.M7.RData')

# retrieving variance components and Highest Density Interval of posterior distributions
VAR['M7','DIC']=fm.M7$fit$DIC

varE=scan("M7_varE.dat")
VAR['M7','residual']= fm.M7$varE
HPD['M7','residual.low']=hdi(varE, ci=0.95)[,2]
HPD['M7','residual.high']=hdi(varE, ci=0.95)[,3]

B.SP=readBinMat('M7_ETA_spat_b.bin')
V.SP=apply(FUN=var,X=(tcrossprod(pc.spat,B.SP)),MARGIN=2)
VAR['M7','spat']=mean(V.SP)
HPD['M7','spat.low']=hdi(V.SP, ci=0.95)[,2]
HPD['M7','spat.high']=hdi(V.SP, ci=0.95)[,3]

B.FAM=readBinMat('M7_ETA_family_b.bin')
V.FAM<-apply(FUN=var,X=(tcrossprod(XFAM,B.FAM)),MARGIN=2)
VAR['M7','family']=mean(V.FAM)
HPD['M7','family.low']=hdi(V.FAM, ci=0.95)[,2]
HPD['M7','family.high']=hdi(V.FAM, ci=0.95)[,3]

B.SNP=readBinMat('M7_ETA_SNP_b.bin')
V.SNP<-apply(FUN=var,X=(tcrossprod(pc.snp,B.SNP)),MARGIN=2)
VAR['M7','snp']=mean(V.SNP)
HPD['M7','snp.low']=hdi(V.SNP, ci=0.95)[,2]
HPD['M7','snp.high']=hdi(V.SNP, ci=0.95)[,3]

B.MET=readBinMat('M7_ETA_MET_b.bin')
V.MET=apply(FUN=var,X=(tcrossprod(pc.met,B.MET)),MARGIN=2)
VAR['M7','met']=mean(V.MET)
HPD['M7','met.low']=hdi(V.MET, ci=0.95)[,2]
HPD['M7','met.high']=hdi(V.MET, ci=0.95)[,3]

B.GE=readBinMat('M7_ETA_GE_b.bin')
V.GE=apply(FUN=var,X=(tcrossprod(pc.ge,B.GE)),MARGIN=2)
VAR['M7','ge']=mean(V.GE)
HPD['M7','ge.low']=hdi(V.GE, ci=0.95)[,2]
HPD['M7','ge.high']=hdi(V.GE, ci=0.95)[,3]

B.METxGE=readBinMat('M7_ETA_METxGE_b.bin')
V.METxGE=apply(FUN=var,X=(tcrossprod(pc.mXg,B.METxGE)),MARGIN=2)
VAR['M7','mXg']=mean(V.METxGE)
HPD['M7','mXg.low']=hdi(V.METxGE, ci=0.95)[,2]
HPD['M7','mXg.high']=hdi(V.METxGE, ci=0.95)[,3]

B.GExC=readBinMat('M7_ETA_GExC_b.bin')
V.GExC<-apply(FUN=var,X=(tcrossprod(pc.geXc,B.GExC)),MARGIN=2)
VAR['M7','geXc']=mean(V.GExC)
HPD['M7','geXc.low']=hdi(V.GExC, ci=0.95)[,2]
HPD['M7','geXc.high']=hdi(V.GExC, ci=0.95)[,3]

B.GExD=readBinMat('M7_ETA_GExD_b.bin')
V.GExD<-apply(FUN=var,X=(tcrossprod(pc.geXd,B.GExD)),MARGIN=2)
VAR['M7','geXd']=mean(V.GExD)
HPD['M7','geXd.low']=hdi(V.GExD, ci=0.95)[,2]
HPD['M7','geXd.high']=hdi(V.GExD, ci=0.95)[,3]

# save the output
write.table(VAR,file='var_spat_HPD.dat')
write.table(HPD,file='HPD_spat.dat')
```

**Evaluating prediction ability**

The following code shows how prediction ability of omic models were calculated

**Models’ definition**

nIter=150000 #number of iteriations

burnIn=20000 #burn in

thin=20

verbose=F

nCV = 20 #number of cross-validation

nFolds = 120 # number of folds to obtain leave-one-family-out

**Matrices to collect the output**

```
YHat=matrix(nrow=n,ncol=4)
mod<-c('M2','M3','M4','M5','M6','M7')
YHat=matrix(nrow=length(y),ncol=length(mod)+2,NA)
colnames(YHat)=c('y','folds',mod)
rownames(YHat)=rownames(TRAIT)

#make matrix for yfix
fix=matrix(nrow=length(y),ncol=length(mod)+2,NA)
rownames(fix)=rownames(TRAIT)
colnames(fix)=c('y','folds',mod)

YHat[,'y']=y[,”trait”]
fix[,'y'] =y[,”trait”]

CORc=matrix(nrow=nCV,ncol=length(mod),NA)
colnames(CORc)=mod
rownames(CORc)=paste0('CV-',1:nCV)

CORd=CORc
```

**Cross-validation**

```
pdf(paste0('crossValidation_XY.pdf'))
for(h in 1:nCV){ # loop over cross-validations

  YHat[,1]=y[,”trait”]
  fix[,1]=y[,”trait”]

  # creating folds with treatment and replicate within the same fold
  library(groupdata2)
  ID=TRAIT[,c(1,2,5)]
  kk <- fold(ID, nFolds, id_col = 'short_id', method = 'n_rand')
  data <- kk[order(kk$ID),]
  folds = data$.folds
  TRAIT$folds<-folds
  YHat[,2]=folds
  fix[,2]=folds

  cat('= CV Number:',h,'=================================\n')
  for(i in 1:nFolds){ # loop over folds
    tst<-which(folds==i)
    #yNA<-y[,”trait”]
    #yNA[tst]<-NA

    yTRN=y[-tst]   ; yTST=y[tst]

    TRT.TRN=as.matrix(XTRT[-tst,]) ; TRT.TST=as.matrix(XTRT[tst,])
    FAM.TRN=XFAM[-tst,] ; FAM.TST=XFAM[tst,]
    SP.TRN=pc.spat[-tst,] ; SP.TST=pc.spat[tst,]
    SNP.TRN=pc.snp[-tst,] ; SNP.TST=pc.snp[tst,]
    MET.TRN=pc.met[-tst,] ; MET.TST=pc.met[tst,]
    GE.TRN=pc.ge[-tst,] ; GE.TST=pc.ge[tst,]
    GExC.TRN=pc.geXc[-tst,] ; GExC.TST=pc.geXc[tst,]
    GExD.TRN=pc.geXd[-tst,] ; GExD.TST=pc.geXd[tst,]
    METxGE.TRN=pc.mXg[-tst,] ; METxGE.TST=pc.mXg[tst,]

    ETA=list(
      trt=list(X=TRT.TRN,model='FIXED'),
      family=list(X=FAM.TRN,model='BRR',saveEffects=T),
      spat=list(X=SP.TRN,model='BRR',saveEffects=T),
      SNP=list(X=SNP.TRN,model='BRR',saveEffects=T),
      MET=list(X=MET.TRN,model='BRR',saveEffects=T),
      GE=list(X=GE.TRN,model='BRR',saveEffects=T),
      GExC=list(X=GExC.TRN,model='BRR',saveEffects=T),
      GExD=list(X=GExD.TRN,model='BRR',saveEffects=T),
      METxGE=list(X=METxGE.TRN,model='BRR',saveEffects=T)
    )

    cat('   Fold:',i,'\n')
    for(j in 1:length(models)){  # loop over models


      # model for fixed effect, random line and spatial effect, and genomic information
      fm.M2 = BGLR(y = yTRN, ETA = ETA[c('trt', 'spat', 'family', 'SNP')], nIter = nIter, burnIn = burnIn, thin = thin, verbose = verbose, df0 = 0.0001, R2 = 0.8)

      rHat_2<-(SNP.TST%*%fm.M2$ETA$SNP$b)
      YHat[tst,'M2']=rHat_2

      fxTST=TRT.TST%*%fm.M2$ETA$trt$b
      fix[tst,'M2']=yTST-fxTST

      # model for fixed effect, random line, spatial and 2 omics
      fm.M3 = BGLR(y = yTRN, ETA = ETA[c('trt', 'spat', 'family', 'SNP', 'MET')],  nIter = nIter ,burnIn = burnIn,  thin = thin, verbose = verbose, df0 = 0.0001, R2 = 0.8)

      rHat_3<-(SNP.TST%*%fm.M3$ETA$SNP$b)+(MET.TST%*%fm.M3$ETA$MET$b)
      YHat[tst,'M3']=rHat_3

      fxTST=TRT.TST%*%fm.M3$ETA$trt$b
      fix[tst,'M3']=yTST-fxTST


      # model for fixed effect, random line, spatial and 2 omics
      fm.M4 = BGLR(y = yTRN, ETA = ETA[c('trt', 'spat', 'family', 'SNP', 'GE')], nIter = nIter ,burnIn = burnIn,  thin = thin, verbose = verbose, df0 = 0.0001, R2 = 0.8);

      rHat_4<-(SNP.TST%*%fm.M4$ETA$SNP$b)+(GE.TST%*%fm.M4$ETA$GE$b);
      YHat[tst,'M4']=rHat_4

      fxTST=TRT.TST%*%fm.M4$ETA$trt$b
      fix[tst,'M4']=yTST-fxTST

      # model for fixed effect, random line, spatial and 3 omics
      fm.M5 = BGLR(y = yTRN, ETA = ETA[c('trt', 'spat', 'family', 'SNP', 'MET', 'GE')], nIter = nIter ,burnIn = burnIn,  thin = thin, verbose = verbose, df0 = 0.0001, R2 = 0.8)

      rHat_5<-(SNP.TST%*%fm.M5$ETA$SNP$b) + (GE.TST%*%fm.M5$ETA$GE$b) + (MET.TST%*%fm.M5$ETA$MET$b)
      YHat[tst,'M5']=rHat_5

      fxTST=TRT.TST%*%fm.M5$ETA$trt$b
      fix[tst,'M5']=yTST-fxTST

      #model for fixed effect, random line, spatial, 3 omics and their interactions
      fm.M6 = BGLR(y = yTRN, ETA = ETA[c('trt', 'spat', 'family', 'SNP', 'MET', 'GE', 'METxGE')], nIter = nIter ,burnIn = burnIn,  thin = thin, verbose = verbose, df0 = 0.0001, R2 = 0.8)

      rHat_6<-(SNP.TST%*%fm.M6$ETA$SNP$b) + (GE.TST%*%fm.M6$ETA$GE$b) + (MET.TST%*%fm.M6$ETA$MET$b) + (METxGE.TST%*%fm.M6$ETA$METxGE$b)
      YHat[tst,'M6']=rHat_6

      fxTST=TRT.TST%*%fm.M6$ETA$trt$b
      fix[tst,'M6']=yTST-fxTST

      #model for fixed effect, random line, spatial, 3 omics, their interactions and treatment effect on transcriptomic data
      fm.M7 = BGLR(y = yTRN, ETA = ETA[c('trt', 'spat', 'family', 'SNP', 'MET', 'GE', 'GExC', 'GExD', 'METxGE')], nIter = nIter ,burnIn = burnIn,  thin = thin, verbose = verbose, df0 = 0.0001, R2 = 0.8)

      rHat_7<-(SNP.TST%*%fm.M7$ETA$SNP$b) + (GE.TST%*%fm.M7$ETA$GE$b) + (MET.TST%*%fm.M7$ETA$MET$b) + (METxGE.TST%*%fm.M7$ETA$METxGE$b) + (GExC.TST%*%fm.M7$ETA$GExC$b) + (GExD.TST%*%fm.M7$ETA$GExD$b)
      YHat[tst,'M7']=rHat_7

      fxTST=TRT.TST%*%fm.M7$ETA$trt$b
      fix[tst,'M7']=yTST-fxTST

      cat('     Model:',models[j],'\n')
      unlink('cv*.dat')
    }
  }
save(YHat, fix, file=paste0('04_PRED_CV',h,'.RData'))

#calculate correlations for each treatment separately

  Index <- grep("^1_*", rownames(YHat))
  YHat_c <- YHat[Index, ]
  fix_c <-fix[Index, ]
  y_c<-y[Index]

  for(j in 1:length(mod)){
    CORc[h,j] = cor(fix_c[,mod[j]], YHat_c[,mod[j]], method = c("pearson"), use="complete.obs")
    Title = paste('CONTROL CV-',h,'(Model',mod[j],') Pearson Correlation=', round(CORc[h,j],3))
    plot(y_c~YHat_c[,mod[j]], main=title, ylab='Observed phenotype', xlab='Predicted phenotype',col=2,cex=.5)
    abline(a=0,b=1,col=4)
  }

  Index <- grep("^2_*", rownames(YHat))
  YHat_d <- YHat[Index, ]
  fix_d <-fix[Index, ]
  y_d<-y[Index]

  for(j in 1:length(mod)){
    CORd[h,j] = cor(fix_d[,mod[j]], YHat_d[,mod[j]], method = c("pearson"), use="complete.obs")
    Title = paste('DROUGHT CV-',h,'(Model',mod[j],') Pearson Correlation=', round(CORd[h,j],3))
    plot(y_d~YHat_d[,mod[j]], main=title, ylab='Observed phenotype', xlab='Predicted phenotype', col=2,cex=.5)
    abline(a=0,b=1,col=4)
  }
}
dev.off()

CORc=rbind(CORc,colMeans(CORc, na.rm = T))
CORc=rbind(CORc,apply(CORc, 2, sd))
rownames(CORc)<-c(paste0('CV-',1:nCV),'Average','SD')
print(round(CORc,3))

CORd=rbind(CORd,colMeans(CORd, na.rm = T))
CORd=rbind(CORd,apply(CORd, 2, sd))
rownames(CORd)<-c(paste0('CV-',1:nCV),'Average','SD')
```
