# Supplementary material for: Relative importance of genotype, gene expression, and DNA methylation on complex traits in perennial ryegrass
Source: Plant Genome. 2022 Aug 17;15(4):e20253. doi: 10.1002/tpg2.20253 (PMC12807231; doi:10.1002/tpg2.20253)
Supplement: Supplementary file 2 [file TPG2-15-e20253-s002.docx]

**Supplemental Material**

**Relative importance of genotype, gene expression and DNA methylation on complex traits in perennial ryegrass**

Marta Malinowska^*^, Anja Karine Ruud^*^, Just Jensen, Simon Fiil Svane, Abraham George Smith , Andrea Bellucci, Ingo Lenk, Istvan Nagy, Mattia Fois, Thomas Didion, Kristian Thorup-Kristensen, Christian Sig Jensen, Torben Asp

Number of pages: 8

Number of Files: 1 (upon submission)

Number of Figures: 5

Supplemental Figure S1. Volumetric water content (VWC) measurements between April and August across two beds. The subplots to (**a**) represent the mean VWC of three soil layers in the middle of each bed represented by different colours with the corresponding standard error shown as a grey shade (n_50cm_=8, n_100cm_=4, n_150cm_=4). The subplot (b) shows the mean VWC data where solid lines with different colours represent the mean soil water content at nine locations with sensors installed 25 cm above the subsurface irrigation system (n=4). The grey shade represents the minimum and maximum water content measured by all 36 sensors. Field capacity (FC) and permanent wilting point (PWP) are shown as dashed blue and red lines, respectively

Supplemental figure S2 Correlation plot between segmented skeleton length (x-axis) from the CNN and manual grid count (y-axis)

Supplemental Figure S3. Combined trace plots showing the distribution of the values of each parameter in the chain for the full model predicting a) DW; b) DMDig; c) CEL, and d) DRL

**a**

**b**

**c**

**d**

Supplemental Figure S4. Inflation of prediction for the above-ground trait. The y‑axis is the regression coefficient of predicted on observed values; the x‑axis is the model number. Horizontal line is regression coefficient of 1. In a situation of no inflation, the regression coefficient for each model should not be significantly different from 1. Based on a t-test, ‘NS’ (not significant) and ‘*’ (p < 0.01) indicate whether coefficient is significantly different from 1 or not.

Supplemental Figure S5. Inflation of prediction for the deep root length. The y‑axis is the regression coefficient of predicted on observed values; the x‑axis is the model number. Horizontal line is regression coefficient of 1. In a situation of no inflation, regression coefficient for each model should not be significantly different from 1. Based on a t-test, ‘NS’ (not significant) and ‘*’ (p < 0.01) indicate whether coefficient is significantly different from 1 or not.
